# Wide Linearity Range and Rapid‐Response Tactile Sensor Inspired by Parallel Structures

**DOI:** 10.1002/advs.202506783

**Published:** 2025-06-29

**Authors:** Weihua Gao, Jiantao Yao, Xianhe Yu, Guoliang Ma, Dakai Wang, Hongnian Yu, Zhiwu Han, Luquan Ren

**Affiliations:** ^1^ Parallel Robot and Mechatronic System Laboratory of Hebei Province School of Mechanical Engineering Yanshan University Qinhuangdao Hebei 066000 China; ^2^ Key Laboratory of Bionic Engineering Ministry of Education Jilin University Changchun Jilin 130022 China; ^3^ School of Computing, Engineering & the Built Environment Edinburgh Napier University Edinburgh Scotland EH10 5DT UK

**Keywords:** fast response and recovery time, force‐electric response regulation, multi‐axis torque decoupling, tactile sensor, wide linear range

## Abstract

The synergistic enhancement of both linear range and response speed is crucial for simplifying the signal processing/conversion of tactile sensors and improving real‐time perception. However, traditional tactile sensors face challenges in quantitatively controlling force‐electrical response and viscoelastic hysteresis, limiting their linear sensing range and response speed. Inspired by parallel structures, Composite Parallel Tactile Sensors (CPTS‐W and CPTS‐S) is proposed, employing parallel elastomers to regulate deformation precisely. These sensors exhibit a wide sensing range up to 450 kPa, with three linear response regions: 0–50 kPa (sensitivity of 0.0080 kPa^−^¹), 50–98 kPa (sensitivity of 0.0022 kPa^−^¹), and 98–423 kPa (sensitivity of 0.0012 kPa^−^¹), significantly reduce dynamic recovery hysteresis of conductive composites, and enhance rapid response capability (48 ms response time and 39 ms recovery time). With excellent dynamic response characteristics across a wide linear sensing range, the results demonstrate broad applicability in areas such as physiological signal monitoring, complex object shape recognition, and multi‐axis torque decoupling perception in robotics.

## Graphical abstract

Inspired by parallel structures, a composite tactile sensor design is proposed using parallel elastomer integration, developing two novel sensors. This strategy optimizes the force‐electric response and viscoelastic hysteresis of the conductive composite, achieving a balanced enhancement of linear sensing range and fast response.

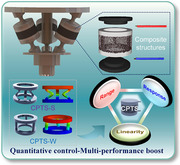

## 1 Introduction

Tactile sensors, which directly convert mechanical stimuli into electrical signals,^[^ ^1^, ^2^, ^3^, ^4^ ^]^ have attracted significant attention due to their potential applications in intelligent control,^[^ ^5^, ^6^, ^7^, ^8^, ^9^, ^10^, ^11^ ^]^ human‐machine interaction,^[^ ^12^, ^13^, ^14^, ^15^, ^16^, ^17^ ^]^ Intent Recognition,^[^ ^18^, ^19^ ^]^ movement monitoring,^[^ ^20^, ^21^, ^22^, ^23^ ^]^ and smart healthcare.^[^ ^24^, ^25^, ^26^, ^27^ ^]^ Among various types of tactile sensors, piezoresistive tactile sensors are particularly promising due to their simple structure, stable signal output, and low fabrication cost.^[^ ^28^, ^29^, ^30^, ^31^, ^32^, ^33^ ^]^ To accommodate multifunctional application scenarios and enable efficient signal processing, piezoresistive tactile sensors should ideally possess both a wide linear sensing range and rapid response capability.^[^ ^34^, ^35^ ^]^ A broad linear sensing range ensures sensitive, consistent signal output over extensive pressure variations, simplifying data processing and signal conversion, while rapid response capabilities facilitate real‐time detection, reducing hysteresis errors and enhancing dynamic tracking performance.

Currently, improvements in linear sensing range and response speed of piezoresistive tactile sensors primarily involve conductive material optimization and microstructure design. Regarding conductive material optimization, high‐performance conductive materials such as MXene,^[^ ^6^, ^36^ ^]^ carbon nanotubes,^[^ ^37^, ^38^ ^]^ and metal nanoparticles^[^ ^39^, ^40^, ^41^ ^]^ significantly enhance the electrical conductivity of sensors, enabling noticeable resistance changes even under slight tactile variations, thereby improving response speed. However, these conductive materials tend to form saturated conductive pathways under high stress, causing resistance changes to stabilize and limiting the sensor's linear operating range. In terms of microstructure design, various microstructures—such as microporous,^[^ ^42^, ^43^ ^]^ micro‐pillar,^[^ ^44^, ^45^ ^]^ micro‐pyramid,^[^ ^11^, ^46^, ^47^ ^]^ micro‐hemisphere,^[^ ^48^, ^49^ ^]^ micro‐wrinkled,^[^ ^50^, ^51^ ^]^ and hierarchical microstructures^[^ ^52^, ^53^ ^]^ have been extensively studied. These microstructures enable the rapid formation of new conductive pathways under slight tactile inputs, making resistance changes more sensitive, improving response speed, and maintaining stable conductive pathway variations over a broad deformation range, thereby enhancing the linear response range. However, long‐term usage may lead to irreversible deformation or collapse of the microstructures, reducing sensitivity and compromising sensor linearity stability. Additionally, certain microstructures exhibit rebound hysteresis after external forces are removed, which negatively impacts the sensor's dynamic response capability. Overall, while extensive research has been conducted to enhance sensor performance through high‐performance conductive materials and microstructure design, inherent material limitations and design constraints hinder quantitative control over force‐electrical response characteristics and viscoelastic hysteresis, preventing the synergistic enhancement of the linear sensing range and response speed in piezoresistive tactile sensors.^[^ ^54^ ^]^

Fortunately, parallel structures exhibit unique advantages in achieving quantitatively controllable deformation. A typical parallel structure consists of multiple branches simultaneously connected to a moving platform and a fixed platform, allowing for precise motion regulation through coordinated actuation.^[^ ^55^, ^56^, ^57^ ^]^ These structures provide controllable mechanical constraints and high structural tunability,^[^ ^58^, ^59^, ^60^ ^]^ enabling refined adjustment of deformation behavior and equivalent stiffness in conductive composites.

In contrast to conventional microstructure‐based designs—which typically rely on microscale features (e.g., pillars, pyramids, or porous networks) and often involve complex fabrication processes—parallel elastomer structures are characterized by macroscale dimensions (above sub‐millimeter level), simple fabrication, and the ability to modulate overall deformation through branch rotation. This allows for integrated tuning of sensitivity, linearity, and sensing range in piezoresistive tactile sensors, offering a promising and novel strategy for achieving both high performance and design flexibility.

In this study, we propose a quantitative regulation mechanism for the force‐electrical response and rebound characteristics of conductive composites using parallel elastomers. Based on this mechanism, two types of Composite Parallel Tactile Sensors (CPTS) are developed: CPTS‐W and CPTS‐S, which incorporate Wren and Sarrus elastomers, respectively. CPTS‐W integrates the Wren elastomer characterized by a zero Poisson's ratio, maintaining nearly constant lateral dimensions during compression. This design facilitates precise deformation control and contributes to an expanded linear sensing range. In contrast, CPTS‐S employs the Sarrus elastomer exhibiting a negative Poisson's ratio, which induces inward contraction upon compression. This structural behavior enhances the mechanical modulation of the conductive composite, thereby improving both pressure sensitivity and linear response performance. To elucidate the working principles of the proposed sensors, Section 2.1 investigates the interaction between parallel elastomers and conductive composites within the sensor architecture, which reveals the underlying mechanism that enhances sensing performance. Sections 2.2 and 2.3 present a comprehensive experimental evaluation of the sensors. Their performance is systematically tested under varying loading frequencies, long‐term cyclic operations, and rapid loading scenarios to verify reliability, durability, and dynamic response. Furthermore, a structural parameter optimization method is introduced, and a mathematical model is established to quantitatively relate physical design parameters to the sensor's force‐electrical response, enabling parametric tunability of performance. Section 3 demonstrates the applicability of the developed sensors in diverse scenarios, including multimodal physiological signal monitoring, complex object shape recognition, and multi‐axis torque decoupling in robotic systems. Section 4 and the Supporting Information provide detailed descriptions of the sensor fabrication process, experimental setups, microstructural characterization, structural design, and the derivation of the underlying models. The proposed regulation mechanism provides new insights for tactile sensor design and holds great potential for advancing wearable devices, health monitoring, and human‐machine interaction.

## 2 Results and Discussion

### 2.1 Inspiration and Design of the CPTS Structure

**Figure 1:**
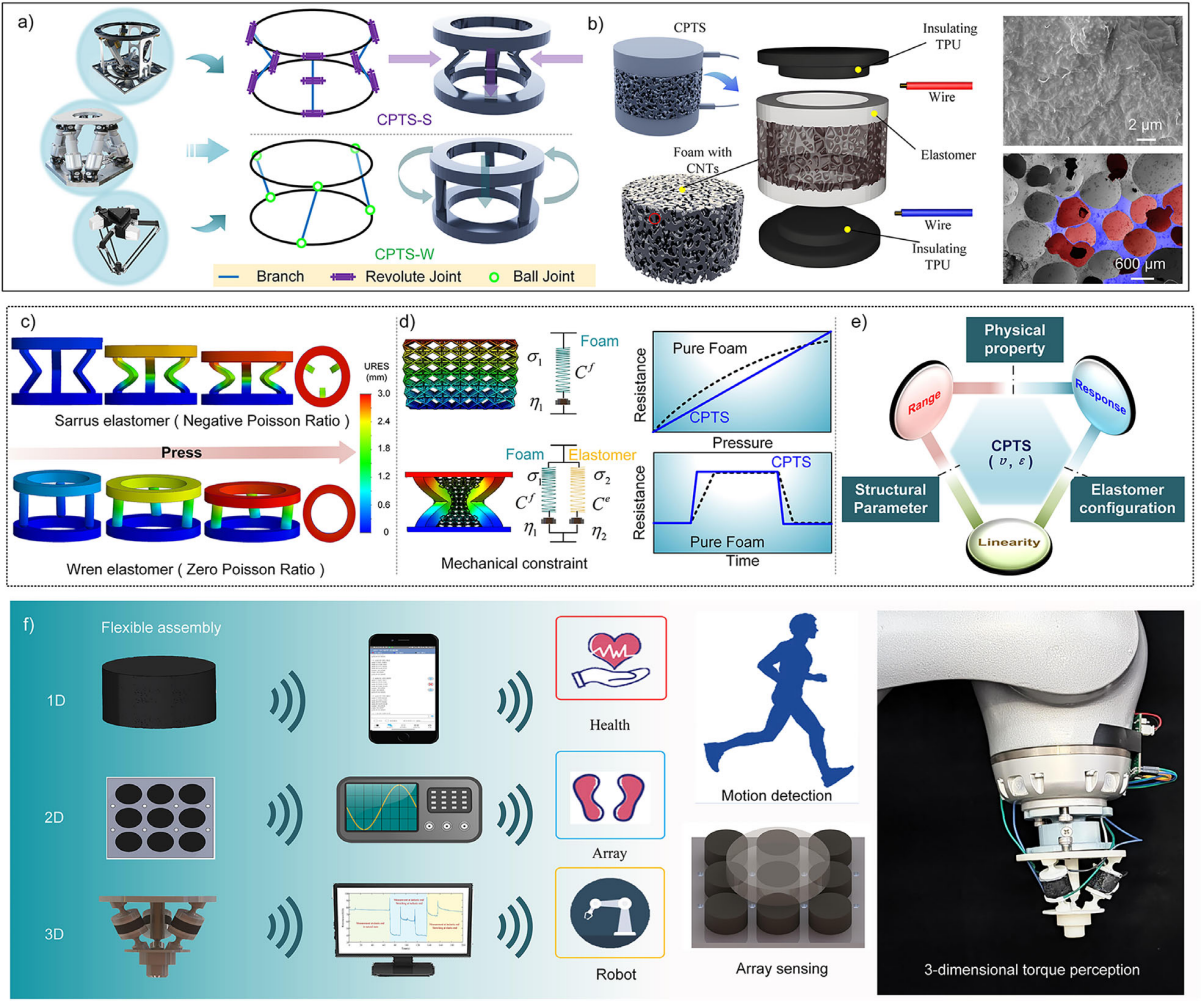
a) Parallel structures‐inspired parallel elastomers. b) Composition of CPTS and its SEM images. c) Simulation of the compression deformation process of parallel elastomers. d) Mechanism analysis of conductive composite material regulation based on parallel elastomers. e) Multi‐performance regulation model based on elastomer structure. f) Potential applications of CPTS in health monitoring, intelligent arrays, and human‐machine interaction.

**Figure** **1**a shows a parallel structure that consists of multiple branches connected to both a moving platform and a fixed platform. Each branch contains multiple kinematic joints, which are actuated by rotary motors or linear actuators, enabling rotational and telescopic motions to achieve precise control of the parallel structure. Compared to commonly used industrial serial robots (such as robotic arms and excavators), parallel structures exhibit higher rigidity, greater load‐bearing capacity, and superior positioning accuracy, making them widely utilized in applications such as precision spacecraft assembly and heavy‐load precision docking. Inspired by the precise motion control and controllable mechanical constraints of parallel structures, we propose two equivalent parallel elastomers—Sarrus elastomer and Wren elastomer—which exhibit negative Poisson's ratio and zero Poisson's ratio characteristics, respectively. Under vertical compression, the Sarrus elastomer exhibits an inward contraction tendency, demonstrating a negative Poisson's ratio. In contrast, the Wren elastomer undergoes branch rotation around the elastomer center under vertical compression, maintaining nearly constant lateral dimensions, thereby exhibiting a zero Poisson's ratio. The deformation processes of the Sarrus elastomer and Wren elastomer under compression are illustrated in Videos S1 and S2 (Supporting Information).

Figure 1b illustrates the structural composition of CPTS, which consists of thermoplastic polyurethane (TPU), wire, elastomer, and CNT‐coated foam. The foam is fabricated using foamed silicone rubber, with CNTs (carbon nanotubes) attached to the pore surfaces, forming a conductive network. Inside the foam, an elastomer made of silicone rubber is embedded. The elastomer is encapsulated by TPU on both upper and lower layers, while wires are secured between the encapsulation layer and the foam to ensure stable signal transmission. Scanning Electron Microscopy (SEM) was used to examine the microstructure of the foam, revealing a highly interconnected 3D porous structure, indicating good permeability and deformation adaptability of the material. The Experimental Section outlines a description of the CPTS fabrication process while the detailed layout is shown in Figure S1 (Supporting Information). Figure S2 (Supporting Information) compares the morphology and pore size of the porous foam before and after graphene attachment, confirming that the graphene coating does not alter the open‐cell structure or pore size, ensuring no adverse effect on the sensor's compressibility. Figure S3 (Supporting Information) shows the elemental composition changes in the foam material before and after CNT attachment, with a significant increase in C element, confirming the effective deposition of CNTs.

Figure 1c presents the 0–3 mm deformation simulation results of the compression processes of the two parallel elastomers, which are cylindrical structures with dimensions of 10 mm in height and 20 mm in diameter. During compression, the Sarrus elastomer exhibits inward contraction of its branches, and the contraction distance can be adjusted by optimizing the structural parameters, enabling a parametrically tunable design for negative Poisson's ratio. In contrast, the Wren elastomer undergoes rotational motion of its branches around the elastomer center during compression, thereby maintaining a constant lateral dimension, exhibiting a zero Poisson's ratio characteristic. The zero/negative Poisson's ratio motion characteristics significantly influence the force‐electric response regulation of the conductive composite material constrained by the elastomer. Figure 1d,e further analyze the regulation mechanism from the perspectives of mechanical principles and electrical sensing characteristics, respectively.

Figure 1d illustrates the compression simulation of both pure foam and the composite material, as well as their spring‐damping equivalent model. The foam material exhibits nonlinear elastic and viscoelastic properties, which can be described using the Kelvin‐Voigt model to represent its stress–strain relationship. The material's nonlinear mechanical characteristics can thus be equivalently modeled as a variable spring‐damping system:

(1) $$\sigma_{1}=C^{f}\;\left(\lambda -1\right)+\eta_{1}\dot{\lambda }$$

where σ_1_ represents the stress of the foam, *C^f^* denotes the elastic modulus of the foam material, η_1_ represents the viscous damping coefficient of the foam, λ is the compression ratio, and $\dot{\lambda }$ denotes the compression rate of the foam.

In the conductive composite material, the foam and elastomer form a parallel mechanical system, where both structures simultaneously bear the load and deform. A modified model of two nonlinear spring‐damping systems is proposed on previous research on the deformation behavior of foam materials,^[^ ^61^ ^]^ which can be expressed as:

(2) $$\sigma =\left(C^{f}+C^{e}\right)\left(\lambda -1\right)+\left(\eta_{1}+\eta_{2}\right)\dot{\lambda }$$

where σ represents the stress of the composite material, *C^e^* denotes the elastic modulus of the elastomer, η_2_ represents the viscous damping coefficient of the elastomer. Equation (2) shows that structural optimization of the elastomer can effectively adjust the mechanical properties of the conductive composite material, thereby enhancing its linear response range. Meanwhile, *C^e^* of the elastomer contributes to improving the recovery capability of the conductive composite, while the η_2_ enhances the energy dissipation efficiency of the system. This enables the sensor to reach a steady state more quickly, reducing hysteresis effects and achieving coordinated regulation of a wide linear sensing range and fast response/recovery capability, as illustrated in Figure 1d.

Figure 1e further elaborates on the performance enhancement mechanism of CPTS from the perspective of its electrical sensing characteristics. The tunable Poisson's ratio (ν) and contraction characteristic (*ε*) of CPTS provide a highly controllable physical foundation for resistance variation in the sensor. To quantitatively describe these key properties, the relationships between ν, *ε*, and sensor performance parameters are established.

(3) $$P_{t}=k_{e}\;\left(\nu ,\varepsilon \right)\cdot V_{e}\left(\nu ,\varepsilon \right)$$

where *P_t_* represents the maximum pressure the sensor can withstand, which is influenced by the equivalent stiffness (*k_e_*) and equivalent volume (*V_e_*) of the material. The ν directly affects the deformation characteristics of the foam. Negative Poisson's ratio materials can increase the contact area upon compression, thereby enhancing the equivalent stiffness and expanding the sensing range. Meanwhile, *ε* reflects the volume change of the material under different pressures, which determines how pressure affects the contact state of CNTs particles. By optimizing ν and *ε*, the mechanical adaptability of the sensor can be improved, and the measurement range can be extended.

(4) $$\frac{dR}{dP}=\alpha \left(\nu ,\varepsilon \right)$$

The linearity describes the relationship between the resistance change of the sensor and the applied pressure. The linearity coefficient (α) is determined by ν and *ε*. Conventional foams tend to exhibit nonlinear deformation during compression, while zero Poisson's ratio materials improve internal deformation behavior. Negative Poisson's ratio materials can further regulate deformation through structural parameter tuning, ensuring a more linear relationship between volume compression and pressure.

(5) $$t=\frac{\eta }{k_{e}\left(\nu ,\varepsilon \right)}\;$$

The response time (*t*) depends on the damping coefficient (*η*) and the equivalent stiffness (*k_e_*), where *k_e_* is influenced by ν. Under the guidance of zero/negative Poisson's ratio elastomers, the conductive composite material undergoes compression in a manner that enhances the rapid formation of conductive paths between CNTs particles, leading to a faster response. Additionally, the rapid rebound of the elastomer affects the deformation recovery speed of the composite material after the external force is removed, thereby impacting the sensor's recovery time.

The microscopic deformation analysis of the conductive medium in pure foam and conductive composite materials during compression is shown in Figure S4 (Supporting Information). As illustrated in Figure 1f, leveraging its exceptional dynamic response performance within a wide linear sensing range, CPTS has been successfully applied in multimodal physiological signal monitoring, complex object shape recognition, and multi‐axis force‐torque decoupling perception for robotics.

### 2.2 Structural Design and Performance Characterization of CPTS‐W

**Figure 2:**
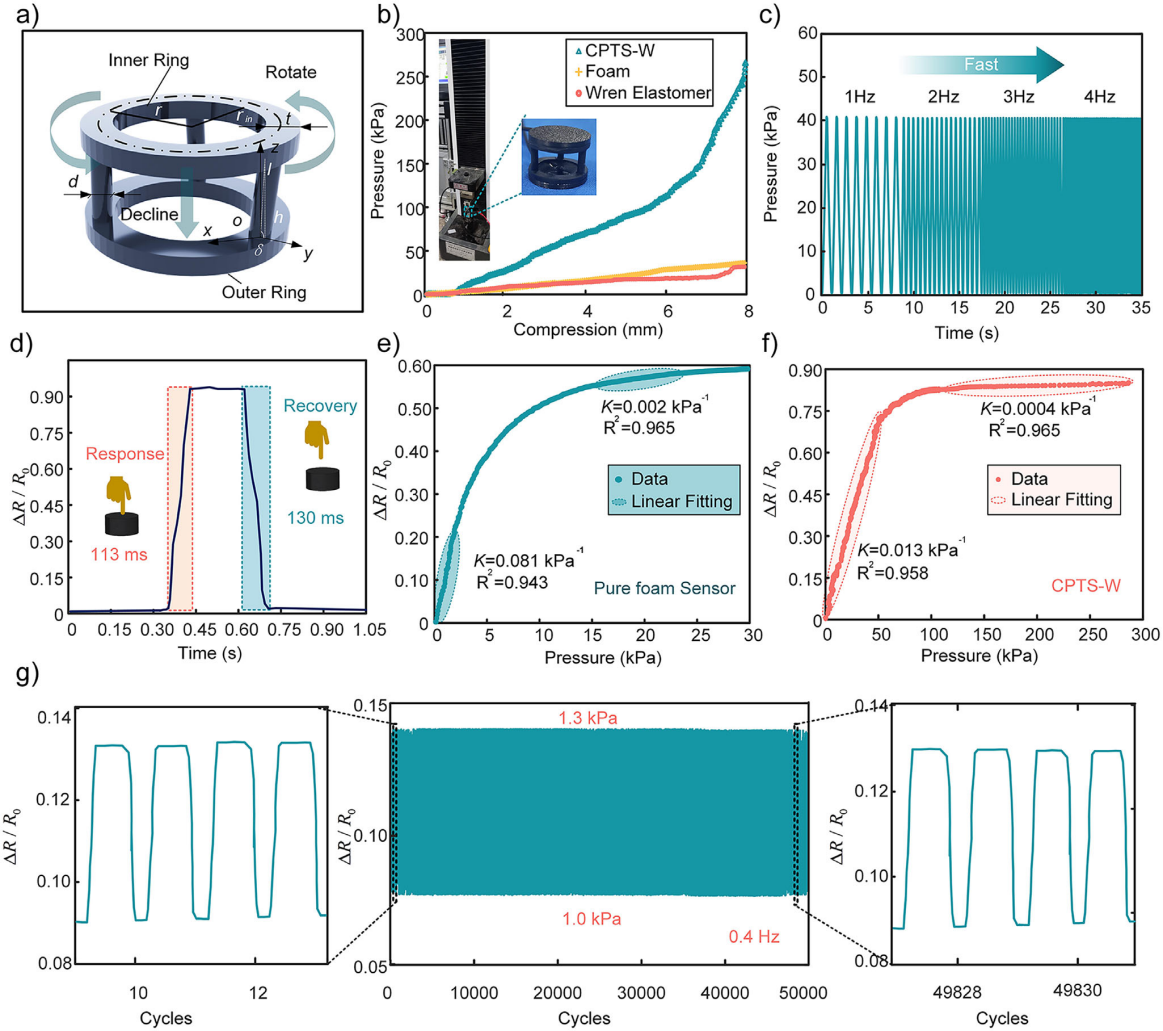
Sensing performance analysis of CPTS‐W. a) Structural parameters of the Wren elastomer. b) Mechanical performance analysis of CPTS‐W under 0–8 mm compression. c) Mechanical response under 0–2.5 mm cyclic compression at frequencies of 1, 2, 3, and 4 Hz. d) Response/recovery time measurement of CPTS‐W. e) Relationship between resistivity change rate and pressure for pure foam sensors during 0–8 mm compression. f) Relationship between resistivity change rate and pressure for CPTS‐W during 0–8 mm compression. g) Resistive stability test of CPTS‐W during 50 000 compression cycles.

**Figure** **2**a illustrates the geometric structure and deformation behavior of the Wren elastomer. During compression, the lower ends of the branches remain fixed, while the upper ends rotate along the upper platform and descend with it. The structural parameters of the Wren elastomer were optimized (details by Note S1 (Supporting Information) in support material), with the finalized parameters listed in Table S1 (Supporting Information). The design ensures that all branches remain confined between the inner ring and outer ring, avoiding interference with the central spatial deformation of the inner ring, thereby achieving a zero Poisson's ratio effect.

The CPTS‐W sensor incorporates the Wren elastomer whose deformation under vertical compression is governed by the rotation and bending of symmetrical branch structures. To establish a closed‐form relationship between the applied external force 𝐹 and the electrical resistance 𝑅, we ultimately derived an explicit expression for the resistance change of the sensor under applied force:

(6) $$\begin{array}{c} R\;\left(F\right)=\frac{h^{2}\pi d_{0}}{A_{0}e^{2}m\lambda }\;{\left(V_{0}\right)}^{\frac{r_{1}}{r_{2}}}\left({\left(A\left(l\cos\delta -\frac{F\cos\delta }{EI}\cdot \frac{l^{3}}{6}\right)\right)}^{-\frac{r_{1}}{r_{2}}}\right)\\ exp\left(\frac{2\lambda d_{0}}{h}\right){\left(V_{0}\right)}^{r_{1}}\left({\left(A\left(l\cos\delta -\frac{F\cos\delta }{EI}\cdot \frac{l^{3}}{6}\right)\right)}^{-r_{1}}\right) \end{array}$$

where *R* represents the sensor's resistance, *h* is Planck's constant, *e* denotes the electron charge, *A*₀ is the initial conductive contact area, m stands for the electron mass, *λ* indicates the insulating barrier potential height, *V*_0_ corresponds to the initial volume of the conductive composite material and *r*_1_, *r*_2_ are constants determined by material properties, particle morphology, and distribution density (measurable through experiments). *E* and *I* represent the elastic modulus and cross‐sectional moment of inertia of the branches. Note S2 (Supporting Information) provides the detailed derivation of the mathematical relationship model between resistance variation and applied force for CPTS‐W.

Figure 2b presents the mechanical performance of CPTS‐W. Under an 8 mm compression displacement, CPTS‐W achieves a pressure of 276.9 kPa, whereas the pure foam only reaches 30 kPa, representing a 9.23‐fold enhancement. This enhancement stems from two factors. On the one hand, the embedded Wren elastomer, with its superior mechanical properties, provides greater resistance to deformation under identical strain. On the other hand, CPTS‐W's zero Poisson ratio enables higher foam compression within the same displacement limit, further amplifying its load‐bearing capacity. These attributes suggest CPTS‐W's potential for a significantly broader sensing range than pure foam‐based sensors.

Figure 2c demonstrates the mechanical response of CPTS‐W under cyclic compression (0–2.5 mm) at frequencies of 1, 2, 3, and 4 Hz, confirming its excellent stability and dynamic performance. Additionally, Figure 2d reveals a rapid response time of 113 ms and a recovery time of 130 ms, highlighting CPTS‐W's high responsiveness. Figure 2e,f compares the resistivity change rate versus pressure for CPTS‐W and pure foam sensors at 8 mm compression. The pure foam sensor exhibits two near‐linear regions: a sensitivity of 0.081 kPa^−^¹ in the 0–3.2 kPa range and 0.002 kPa^−^¹ in the 16.1–23.5 kPa range. CPTS‐W also shows two linear sensing intervals: 0.013 kPa^−^¹ (0–60 kPa) and 0.0004 kPa^−^¹ (116–279 kPa). The Wren elastomer's regulation of composite conductive material deformation endows CPTS‐W with a superior linear sensing range. Figure 2g evaluates the signal stability characterization 50 000 loading cycles performed at a loading frequency of 0.4 Hz within a pressure range of 1.0–1.3 kPa, where CPTS‐W achieves a cyclic error of 0.19%, confirming its long‐term reliability. The calculation methods for sensitivity and cyclic error are detailed (Note. S3, Supporting Information).

### 2.3 Structural Design and Performance Characterization of CPTS‐S

By incorporating the zero Poisson's ratio regulation of the Wren elastomer, CPTS‐W achieves significant improvement in tactile sensor performance. Furthermore, the performance enhancement of CPTS‐S through negative Poisson's ratio regulation based on the Sarrus elastomer is analyzed.

**Figure 3:**
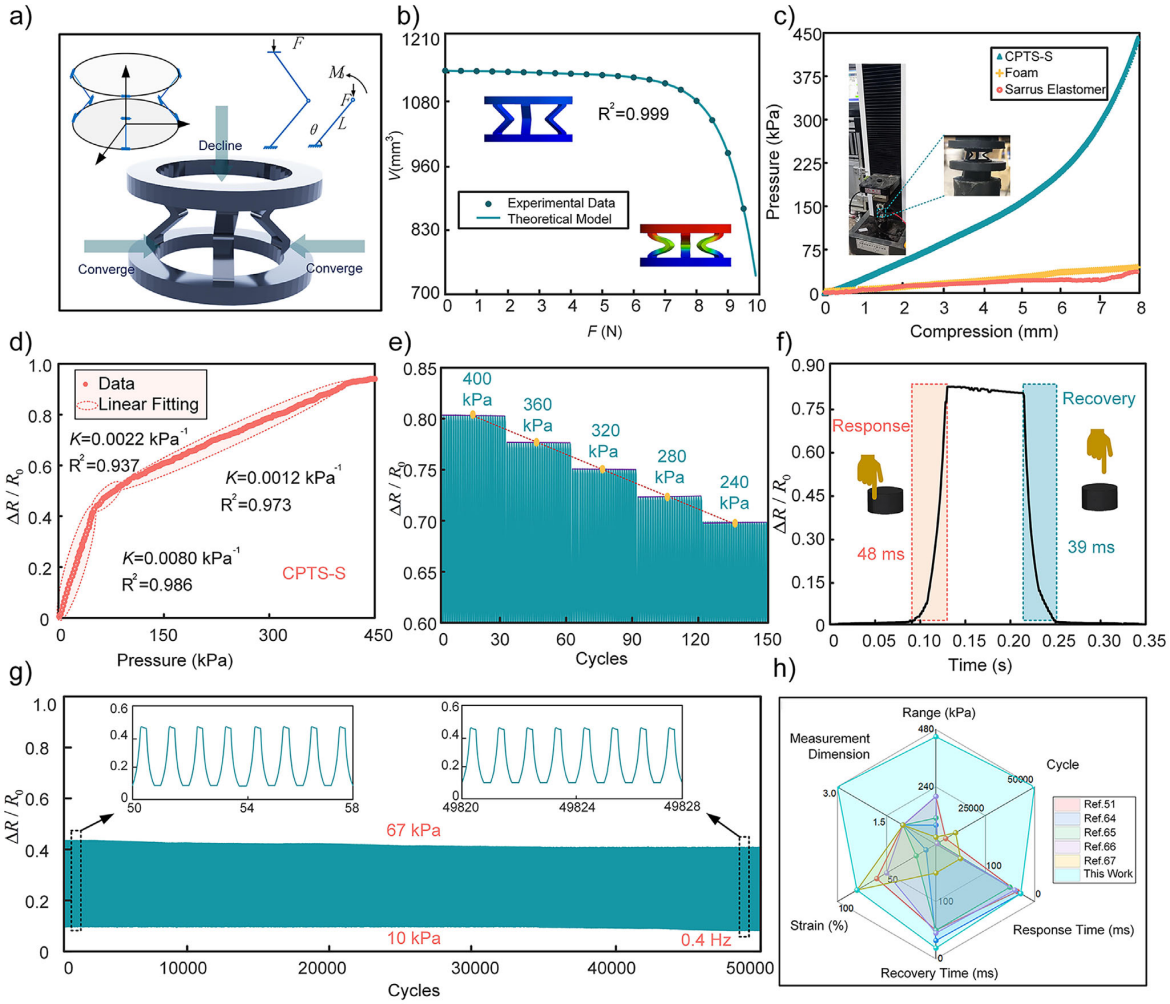
Sensing performance analysis of CPTS‐S. a) Kinematic characterization of Sarrus elastomer. b) Envelope volume variation of Sarrus elastomer under external force. c) Mechanical performance analysis of CPTS‐S during 0–8 mm compression. d) Relationship between resistivity variation rate and pressure for CPTS‐S during 0–8 mm compression. e) Signal linearity stability analysis during cyclic loading (240–400 kPa). f) Response/recovery time measurement of CPTS‐S. g) Resistive stability test of CPTS‐S over 50 000 compression cycles. h) Key performance comparison between CPTS‐S and referenced studies.^[^ ^49^, ^62^, ^63^, ^64^, ^65^ ^]^

Unlike the zero Poisson's ratio characteristic that maintains constant lateral dimensions during compression, the degree of lateral contraction in negative Poisson's ratio structure can be precisely regulated through structural parameters. Note S4 (Supporting Information) provides the detailed derivation of the mathematical relationship model between resistance variation and applied force for CPTS‐S. Based on the structural parameters contained in this model, the sensor's structure was optimally designed, with the final optimization results presented in Table S2 (Supporting Information). **Figure** **3**a illustrates the deformation characteristics of the Sarrus elastomer, where the envelope volume change under pressure can be expressed as:

(7) $$V\left(F\right)=\pi \left(-\frac{Fcos\theta }{24EI}L^{4}+\frac{Fcos\theta }{6EI}L^{3}-\frac{L^{3}}{6EI}\right)$$

where *V* represents the internal volume of the Sarrus elastomer, *F* denotes the applied external force, *θ* is the angle between the platform and branches, L stands for the branch length, while *E* and *I* represent the elastic modulus and cross‐sectional moment of inertia of the branches, respectively. Figure 3b presents both the theoretical results from Equation (7) and the actual deformation of the elastomer during compression. With a regression coefficient (R^2^) of 0.999, these results validate the theoretical model for volume variation in the Sarrus elastomer. Based on this equation, structural parameters can be optimized to quantitatively control the volume change of the Sarrus elastomer under external force *F*. Following the percolation theory, we ultimately derived an explicit expression for the resistance change of the sensor under applied force:

(8) $$\begin{array}{c} R\left(F\right)=\frac{h^{2}\pi d_{0}}{A_{0}e^{2}m\lambda }\;{\left(V_{0}\right)}^{\frac{r_{1}}{r_{2}}}\left(\pi {\left(-\frac{Fcos\theta }{24EI}L^{4}+\frac{Fcos\theta }{6EI}L^{3}-\frac{L^{3}}{6EI}\right)}^{-\frac{r_{1}}{r_{2}}}\right)\\ exp\left(\frac{2\lambda d_{0}}{h}\right){\left(V_{0}\right)}^{r_{1}}\left(\pi {\left(-\frac{Fcos\theta }{24EI}L^{4}+\frac{Fcos\theta }{6EI}L^{3}-\frac{L^{3}}{6EI}\right)}^{r_{1}}\right) \end{array}$$

Equation (8) demonstrates the influence of structural parameters on resistance variation, thereby confirming the Sarrus elastomer's capability to regulate the force‐electrical response of conductive composite materials. Considering both material attributes and manufacturing process characteristics, we have optimized the elastomer's structural parameters (Table S2, Supporting Information).

Figure 3c presents the relationship between compressive displacement and pressure for CPTS‐S, pure foam, and the Sarrus elastomer during 0–8 mm compression. At 8 mm displacement, CPTS‐S withstands a pressure of 450 kPa, which is 15 times higher than that of pure foam under identical conditions and significantly exceeds the 279 kPa achieved by CPTS‐W at the same compression level. This enhanced pressure range stems from the negative Poisson's ratio effect of the Sarrus elastomer: compared to the zero Poisson's ratio behavior of the Wren elastomer, the negative Poisson's ratio regulation induces greater lateral contraction of the foam at equivalent compression heights, thereby generating stronger resistance to deformation. This characteristic simultaneously endows CPTS‐S with a broader sensing range than CPTS‐W.

Figure 3d demonstrates the correlation between resistivity change rate and applied pressure for CPTS‐S during 0–8 mm compression. The sensor exhibits a wide linear sensing range, with three distinct linear response regions: 0–50 kPa (sensitivity of 0.0080 kPa^−^¹), 50–98 kPa (sensitivity of 0.0022 kPa^−^¹), and 98–423 kPa (sensitivity of 0.0012 kPa^−^¹). Compared to both pure foam sensors and CPTS‐W, CPTS‐S achieves substantially expanded linear sensing capabilities across the entire pressure range.

Figure 3e shows the results of 150 cyclic loading tests on CPTS‐S within its 240–400 kPa linear range, where the sensor maintains excellent resistive linearity with consistent gradient variation. Figure 3f reveals response and recovery times of 48 and 39 ms respectively, confirming its rapid dynamic response characteristics.

As shown in Figure 3g, the CPTS‐S sensor maintains excellent stability over 50 000 loading cycles (10–67 kPa) with a minimal cycle error of 0.12%. Compared to previous tactile sensors (Figure 3h), the CPTS‐S exhibits superior performance in terms of sensing range, response speed, and durability. Additionally, Table S3 (Supporting Information) provides a more comprehensive set of quantitative performance indicators compiled from a broader range of related studies, enabling a more rigorous and transparent comparative analysis. As shown in Table S3 (Supporting Information), although certain porous piezoresistive sensors offer high sensitivity in narrow low‐pressure ranges, they often underperform in aspects such as response speed, sensing range, or durability. Due to the physical constraint of ΔR/R₀ < 1 in compressive porous sensors,^[^ ^17^, ^18^, ^19^, ^20^, ^21^, ^22^, ^23^ ^]^ the relative sensitivity is inherently limited. At 80% strain, the CPTS‐S achieves a resistance change of ≈90%, approaching this theoretical limit. Although the relative sensitivity is moderate (0.0012 kPa^−^¹), the CPTS‐S demonstrates an outstanding combination of ultra‐broad pressure range (0–450 kPa), fast response (48/39 ms), and high durability, making it a reliable sensing solution for real‐world applications such as human motion monitoring, collaborative robotics, and human‐machine interaction.

## 3 Applications for CPTS

**Figure 4:**
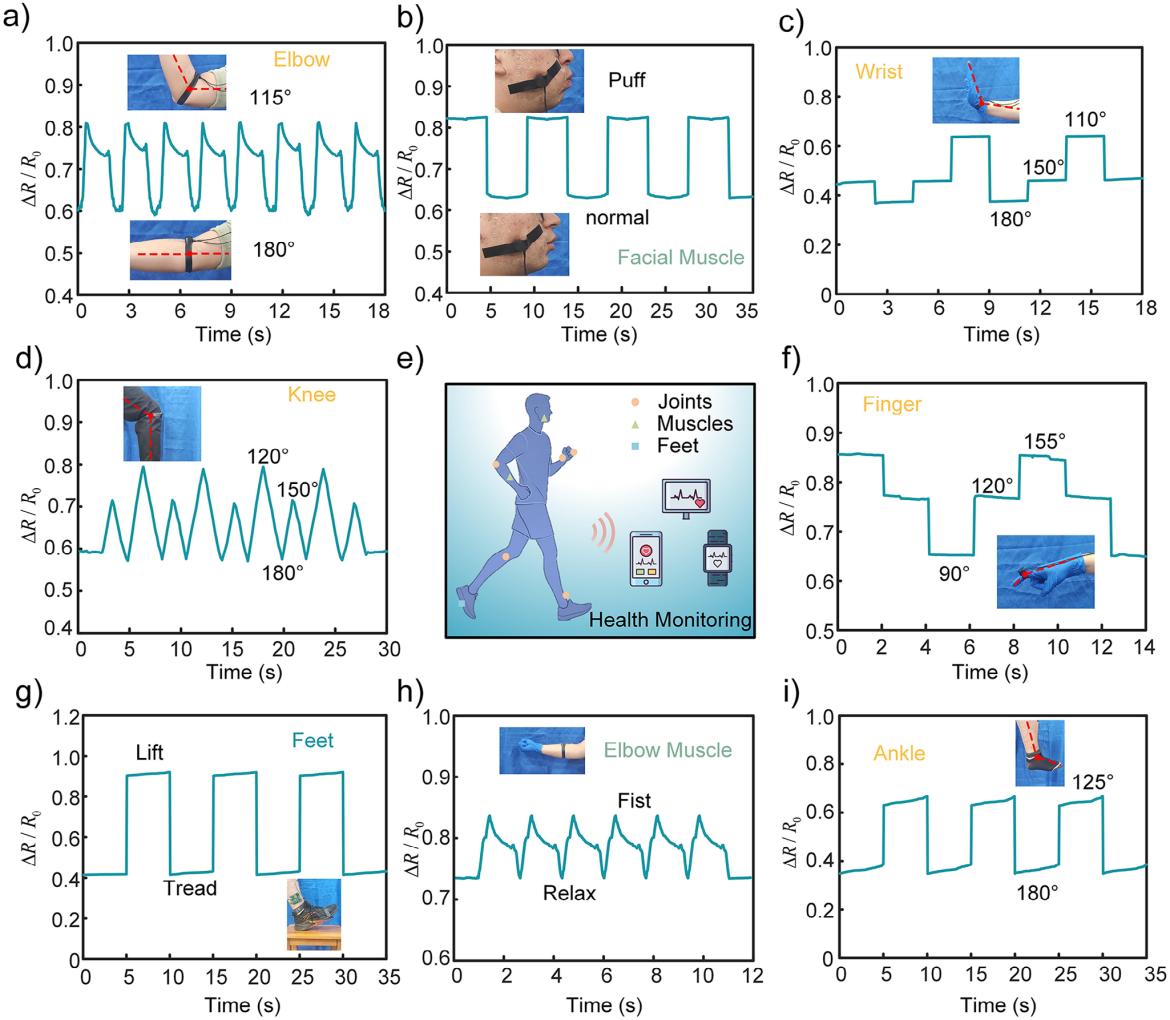
Human‐interactive experiments based on CPTS‐W. a) Elbow flexion measurement. b) Facial muscle monitoring. c) Wrist bending detection. d) Knee flexion measurement. e) Functional classification of interactions. f) Finger bending detection. g) Plantar pressure monitoring. h) Muscle pressure detection during fist clenching.

**Figure** **4** demonstrates the capability of CPTS‐S in monitoring eight different human physiological signals, highlighting its broad application potential in health monitoring, motion feedback, and rehabilitation therapy, with continuous motion monitoring detailed in Video S3 (Supporting Information).

As shown in Figure 4e, CPTS‐S can monitor three distinct physiological types: joints, muscles, and feet. Figure 4a,c,d,f,i illustrates the resistance changes during joint motion monitoring (elbow, wrist, knee, finger, and ankle) at different flexion angles, enabling multi‐axis motion perception for rehabilitation in patients with movement disorders. Figure 4b,h demonstrate applications in facial and forearm muscle monitoring, where muscle exertion data analysis helps clinicians assess muscle fatigue levels and optimize exercise regimens. Leveraging its exceptional sensing range, CPTS‐S enables foot pressure distribution monitoring (Figure 4g), providing critical insights for gait analysis in elderly individuals and fall‐risk detection. These results collectively underscore the sensor's versatility in wearable health monitoring, sports science, and assistive medical diagnostics.

**Figure 5:**
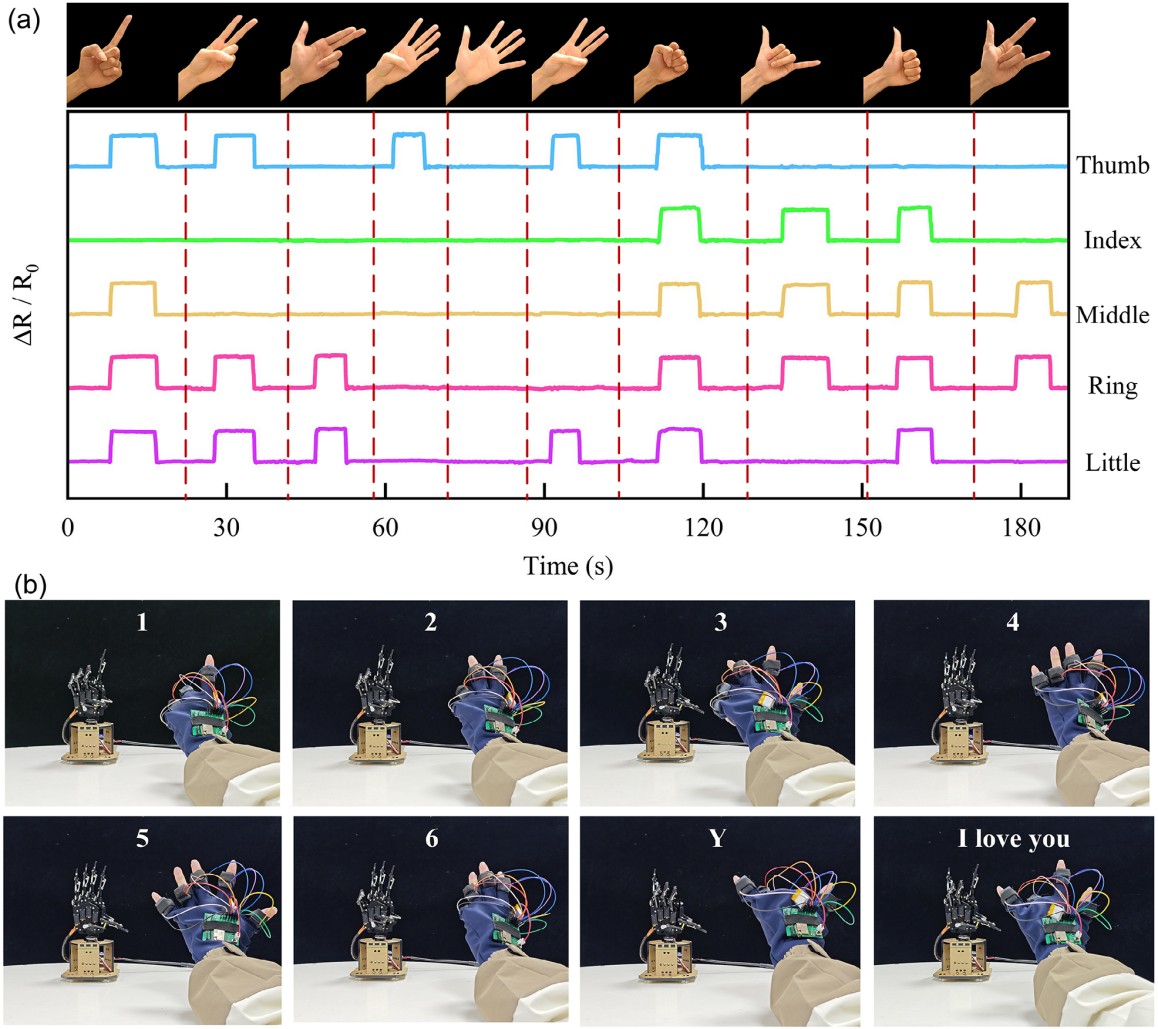
The CPTS‐S in human‐computer interaction to control a robotic hand. a) Relative resistance responses of five fingers during gesture recognition. b) Real‐time robotic hand actuation replicating human gestures based on sensor signals.

**Figure** **5** further extends the application scope of CPTS by demonstrating its feasibility as a human‐machine interface for robotic hand control. In this proof‐of‐concept experiment, five CPTS‐S units were integrated into a wearable glove to detect individual finger motions. As shown in Figure 5a, a series of distinct hand gestures, including numerical gestures (1–6), the Sign Language letter “Y,” and the “I love you” gesture, were performed sequentially. The corresponding relative resistance changes (ΔR/R₀) of the thumb, index, middle, ring, and little fingers were recorded and plotted over time, clearly revealing consistent, repeatable, and distinguishable signal profiles for each gesture.

Figure 5b shows that the signals were subsequently used to actuate a robotic hand in real‐time, with experimental details provided in Video S4 (Supporting Information). The robotic hand accurately replicated each gesture, indicating that the CPTS‐enabled glove could effectively capture and transmit fine‐grained motion information. This experiment highlights the potential of CPTS in next‐generation human‐machine interaction scenarios, such as robotic teleoperation, prosthetic control, and gesture‐based communication systems. The high fidelity of gesture replication, enabled by the sensor's high sensitivity, underscores its promising role in wearable intelligent interfaces and soft robotics.

**Figure 6:**
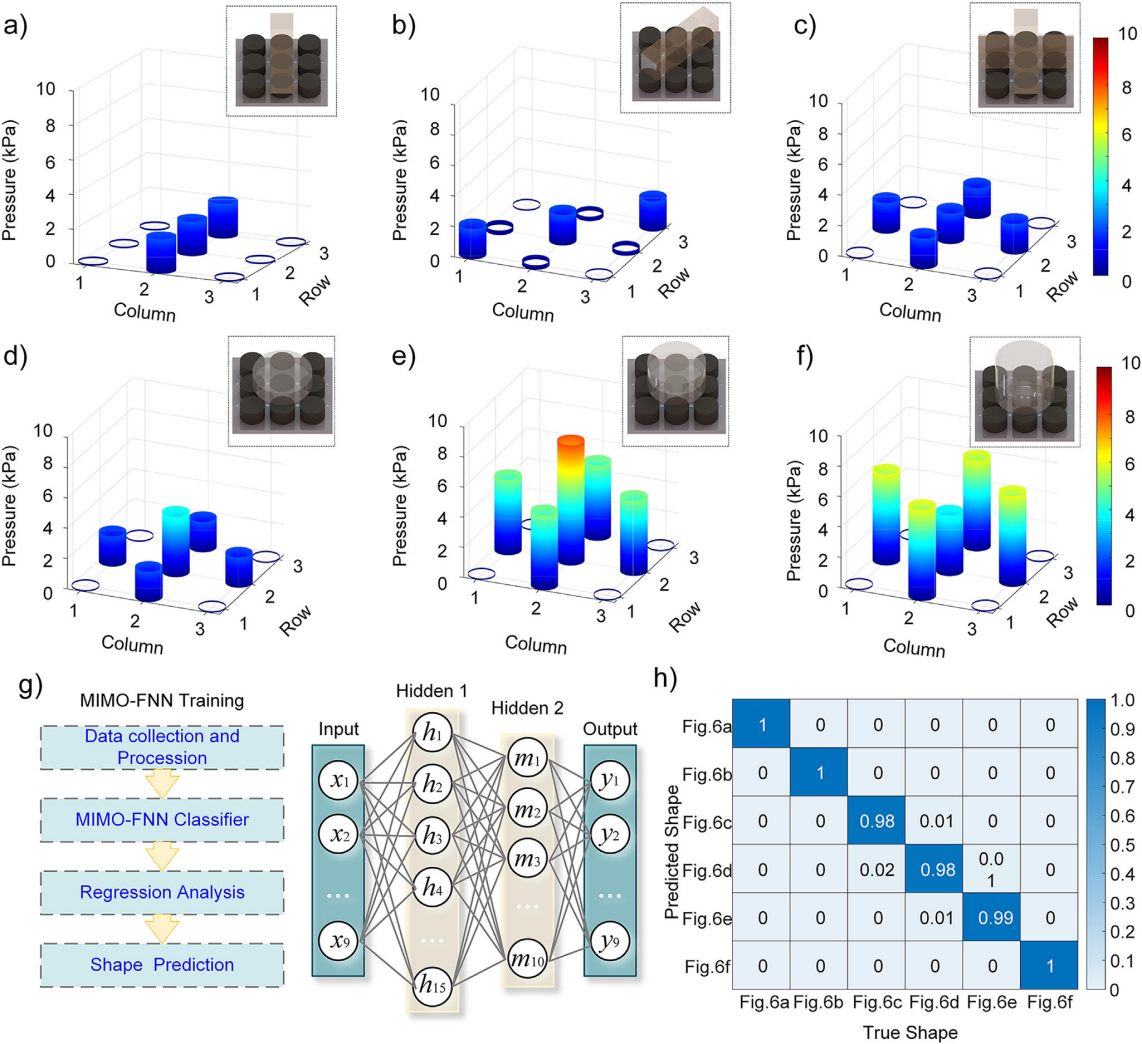
Array sensing based on CPTS‐S. a–f) Pressure distribution histograms of the sensor array for objects with different shapes/weights. g) Multi‐input multi‐output feedforward neural network (MIMO‐FNN) design for pressure prediction using resistance signals. h) Shape prediction results of contact objects.

As shown in **Figure** **6**a–f, a 3 × 3 tactile sensor array was employed to detect both contact geometry and pressure magnitude of objects, with the acquisition system and hardware configuration of the sensing array detailed in Figure S5 (Supporting Information). Notably distinct from existing studies, this sensor array features millimeter‐scale compressibility, granting it unique advantages in identifying dome‐shaped structures Figure 6f shows that when testing a dome‐bottomed object (e.g., a soda can base), the pressure sensor array not only accurately localized contact positions but also clearly revealed pressure differentials between the central zone and peripheral contact regions. Increasing the array density could potentially enable arc‐height prediction through pressure distribution gradients.

Figure 6g presents the detailed architecture of our Multi‐Input Multi‐Output Feedforward Neural Network (**MIMO‐FNN**) for shape recognition, where each input node corresponds to a sensor's resistance data. After processing through two hidden layers, the network outputs predicted pressure values for precise object shape recognition. Prediction results from 3000 shape‐sensing trials (Figure 6h) demonstrated 99.33% average accuracy. These findings confirm that the array achieves not only 2D planar pressure measurement but also exhibits promising capability for 3D topographic reconstruction of specialized dome‐shaped contact surfaces.

**Figure 7:**
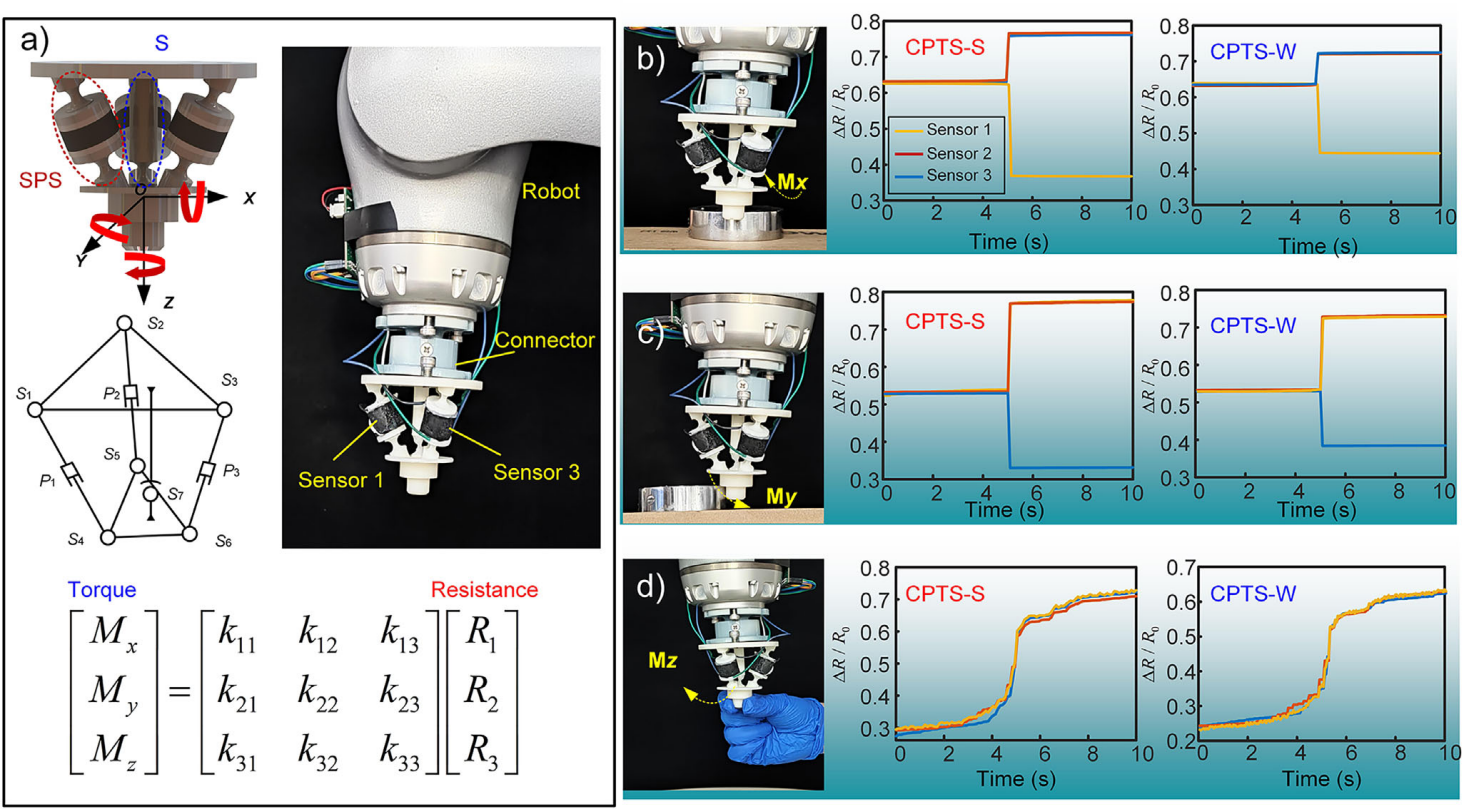
Three‐axis torque sensing measurement based on CPTS. a) Composition of the CPTS‐integrated 3D torque decoupling measurement device and the mapping relationship between torque and resistance. b) Sensor signal variation under Mx torque. c) Sensor signal variation under My torque. d) Sensor signal variation under Mz torque.

**Figure** **7** demonstrates the application of CPTS in three‐axis torque decoupling perception, with experimental details provided in Video S5 (Supporting Information). We develop a specialized 3D torque decoupling measurement device (structural details in Figure S6, Supporting Information) consisting of three *SPS* (spherical‐prismatic‐spherical) limbs and one *S* (spherical) limb (Figure 7a). In this configuration, the *SPS* limbs enable linear motion along their axes while the *S* limb provides rotational freedom about the center point, with the *S* limb completely constraining translational degrees of freedom. Under external torque loading, the *SPS* limbs respond through telescopic motion while the *S* limb adjusts end‐effector orientation, causing integrated CPTS units to generate corresponding compression/tension signals. This innovative design achieves precise 3D torque decoupling through differential signal analysis from the three limbs (kinematic analysis in Note S5, Supporting Information). The mapping relationship between CPTS resistance signals and external torques (Figure 7a) benefits from both the CPTS's exceptional linear sensing range and the mechanism's mechanical decoupling, resulting in constant parameters (*k*₁₁–*k*₃₃) in the mapping matrix that enable direct 3D torque analysis through resistance variations.

Robotic collision tests (Figure 7b,c) using both CPTS‐S and CPTS‐W at 30 mm s^−1^ feed rate demonstrated the system's rapid response capability, where characteristic signal patterns (e.g., increased Sensors 1 and 3 signals with decreased Sensor 2 signal) accurately determined limb displacement, mechanism deflection, and torque magnitude/direction. Furthermore, human‐robot interaction tests (Figure 7d) confirmed CPTS's real‐time torque monitoring capability during physical contact, establishing this integrated solution as an advanced approach for next‐generation robotic safety systems.

## 4 Conclusion

This study presents a novel approach to tactile sensor design based on parallel elastomers, offering precise control over both the electromechanical response and viscoelastic properties of conductive polymer composites. Using this approach, two sensor prototypes—CPTS‐W and CPTS‐S—were developed and characterized. These sensors exhibit a wide linear sensing range with three distinct response regions: 0–50 kPa (sensitivity of 0.0080 kPa^−^¹), 50–98 kPa (sensitivity of 0.0022 kPa^−^¹), and 98–423 kPa (sensitivity of 0.0012 kPa^−^¹). The sensors also feature fast response/recovery times (48/39 ms) and excellent stability (0.12% signal variation over 50 000 cycles). The practical applicability of these sensors was validated through demonstrations in physiological monitoring, object recognition, and robotic torque measurement, highlighting their potential for use in wearable devices, health monitoring systems, and human‐machine interfaces.

While the proposed sensors demonstrate excellent performance in linearity, response speed, and durability, their sensitivity remains a focus for future improvement. The relative resistance variation (ΔR/R₀) in piezoresistive sensors based on porous materials is inherently constrained, limiting sensitivity over a wide pressure range. Despite this, the absolute resistance variation from megaohms to kiloohms allows for reliable signal detection in practical applications such as human motion monitoring and robotics.

The fabrication process involves precise alignment of parallel elastomeric elements, which may present challenges for large‐scale manufacturing. Future efforts will focus on enhancing sensitivity through microstructural engineering, optimizing the fabrication process for scalability, and reducing the device size for compact wearable systems. Additionally, we will continue to refine the equivalent parallel elastomer design mechanism, with the goal of achieving breakthroughs in sensor sensitivity, and miniaturization, and further expanding its applicability in next‐generation tactile sensing systems.

## 5 Experimental Section

### Experimental Material

Silicone rubber (Shore hardness 45) was sourced from HongCheng Materials Company Ltd, China. Conductive TPU materials were provided by YouSu Company Ltd, China. Both the casting molds and conductive TPU components were fabricated using the Bambu Lab A1 3D printer, supplied by Top Bamboo Technology Company (Shenzhen, China). The HY‐F662 two‐component expandable foam silicone rubber was procured from HongCheng Materials Company Ltd, China. A water‐based carbon nanotube (**CNTs**) slurry with a solid content of 13% was obtained from Suzhou Carbon Ink Technology Co., Ltd., China. Sensor fabrication was conducted under controlled temperature and pressure conditions using a DZF‐6050 vacuum drying oven from Shanghai Yiheng Scientific Instrument Co., Ltd.

### Preparation Process

The preparation process of the sensor is illustrated in Figure S1 (Supporting Information). Equal masses (3 g each) of expandable foam silicone A‐Liquid and B‐Liquid were mixed (Figure S1a, Supporting Information) and stirred at 60 rpm for 5 min using a magnetic stirrer to produce a foaming silicone mixture with vulcanization and foaming properties. Figure 1b shows the assembly process of the sensor skeleton. The elastomer was prepared using silicone rubber with the Shore hardness of 45, and insulating TPU with the Shore hardness of 95, along with conductive silver glue, were used for bonding. The assembled structure was then placed in a mold. Next, the foaming silicone mixture was poured into the mold (Figure S1c, Supporting Information) and left to cure in a sealed environment at 25 °C for 12 h, forming the sensor without CNTs. The sensor was subsequently immersed in a CNT solution with a solid content of 13% and kept under 0.1 kPa pressure at 25 °C for 48 h. It was then placed in a drying oven at 120 °C for 8 h. Unattached CNTs and other impurities generated during preparation were removed using ultrasonic cleaning in ethanol. The resulting sensor, shown in Figure S1d (Supporting Information), is a cylindrical structure with dimensions of 10 mm in height and 20 mm in diameter.

### Characterizations and Measurements

The microstructure of the foam materials was observed using a Zeiss Gemini 360 field‐emission scanning electron microscope, and elemental composition mapping of the foam was analyzed with an Oxford Xplore 30 energy dispersive spectrometer. Mechanical performance testing of the sensors was carried out using an FBS tensile testing machine from FURBS Company, while the electrical resistance performance was evaluated using a Keithley DMM6500 6.5‐digit digital multimeter.

## Conflict of Interest

The authors declare no conflict of interest.

## Supporting information



Supporting Information

Supplemental Video 1

Supplemental Video 2

Supplemental Video 3

Supplemental Video 4

Supplemental Video 5

## Data Availability

The data that support the findings of this study are available from the corresponding author upon reasonable request.
