# Supplementary material for: Wide Linearity Range and Rapid‐Response Tactile Sensor Inspired by Parallel Structures
Source: Adv Sci (Weinh). 2025 Jun 29;12(32):e06783. doi: 10.1002/advs.202506783 (PMC12407343; doi:10.1002/advs.202506783)
Supplement: Supplementary file 1 — Supporting Information [file ADVS-12-e06783-s004.docx]

Supporting Information

**Wide Linearity Range and Rapid-Response Tactile Sensor Inspired by Parallel Structures**

*Weihua Gao*, *Jiantao Yao**, *Xianhe Yu*, *Guoliang Ma**, *Dakai Wang*, *Hongnian Yu*, *Zhiwu Han*,*and Luquan Ren*

W．Gao, J. Yao, X. Yu, G. Ma

Parallel Robot and Mechatronic System Laboratory of Hebei Province

School of Mechanical Engineering

Yanshan University

Qinhuangdao, Hebei 066000, China.

E-mail: jtyao@ysu.edu.cn (J. Yao) , magl@ysu.edu.cn (G. Ma)

G. Ma, D. Wang, Z. Han, L. Ren

Key Laboratory of Bionic Engineering, Ministry of Education

Jilin University

Changchun, Jilin 130022, China

H. Yu

School of Computing, Engineering & the Built Environment

Edinburgh Napier University

Edinburgh, Sctoland, EH10 5DT, UK.

Supplementary Figures


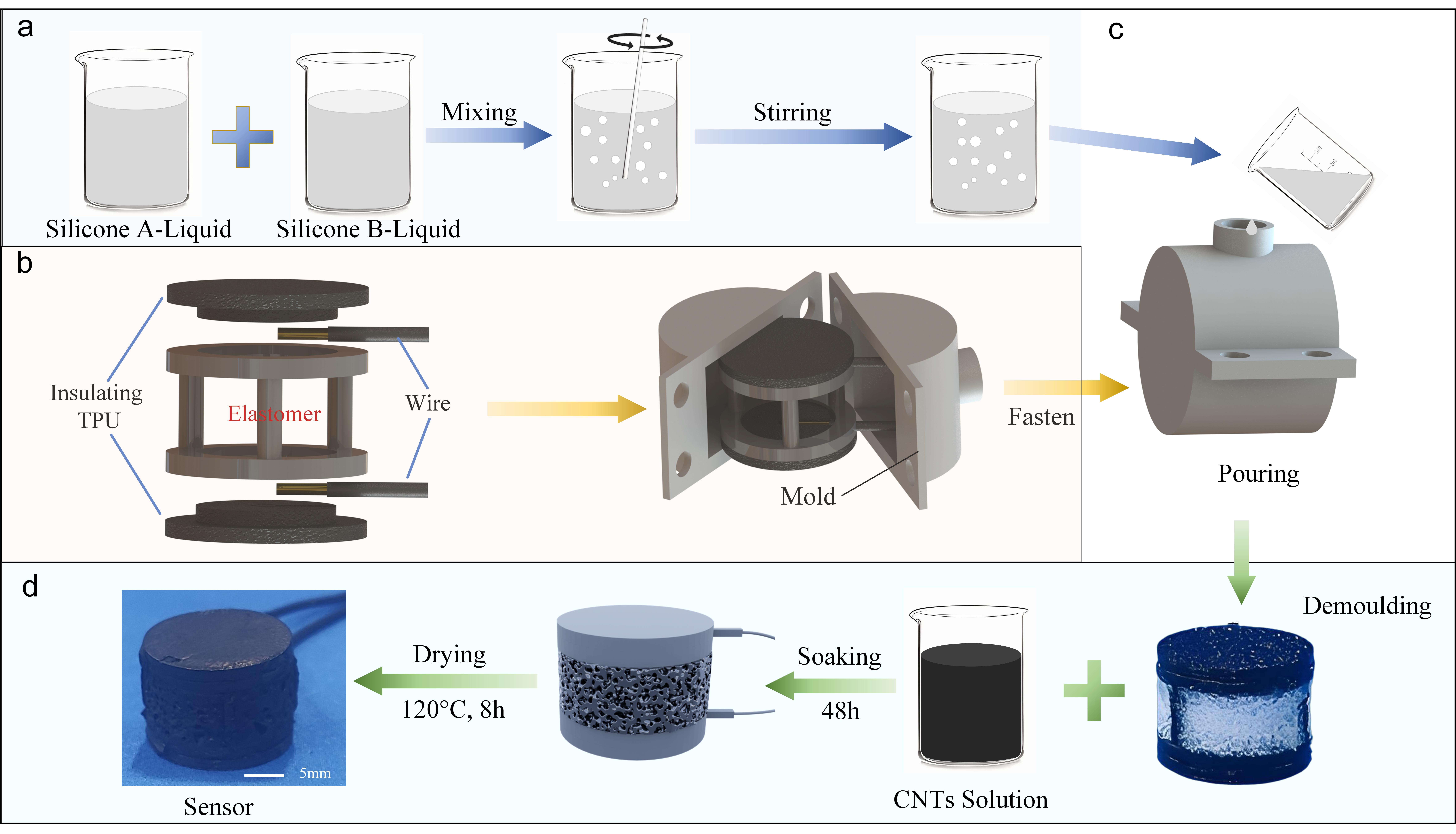


**Figure S1.** Sensor preparation process. a) Preparation of expandable foam silicone rubber mixed solution. b) Sensor skeleton package. c) CNTs attachment treatment.


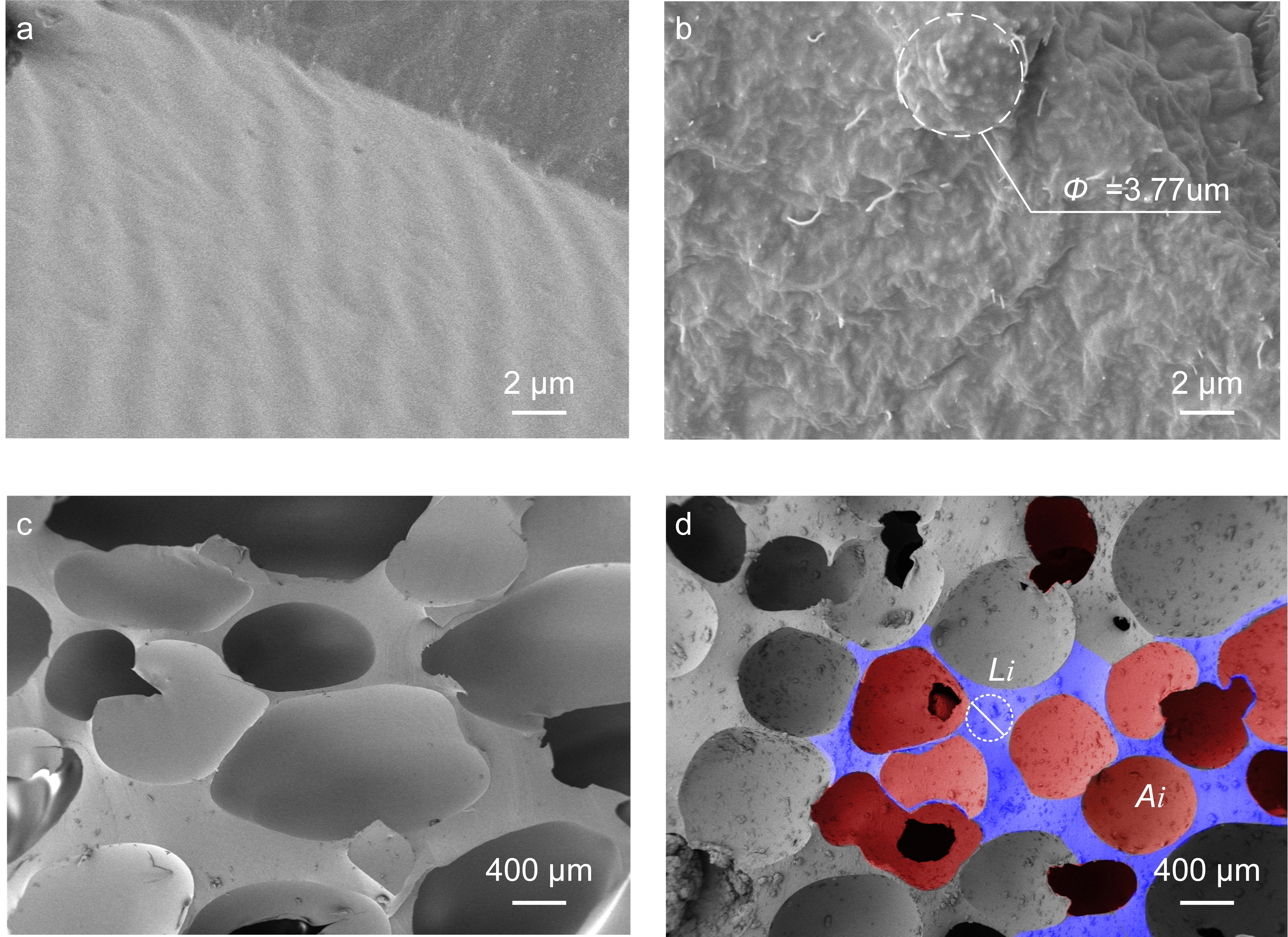


**Figure S2.** Distribution of microscopic surfaces and voids of the foam before and after CNTs were infiltrated.

Figure S2 presents the microscopic images of the porous foam before and after CNTs attachment. As shown in Figures S2a and S2b, the foam surface is smooth prior to CNTs immersion, while the surface becomes rough and wrinkled after CNTs attachment, indicating the influence of CNTs on the foam's microstructure. On the other hand, Figures S2c and S2d demonstrate that the porous macroscopic characteristics of the foam remain unaffected by CNTs attachment, retaining its ability to endure large-scale compressive deformation.

Furthermore, a detailed statistical analysis of the pore diameter *L_i_* and area *A_i_* of the porous foam was conducted. Based on a dataset of 600 measurement data, the average pore diameter and area before and after CNT immersion were 332.05 µm (0.48 mm²) and 339.58 µm (0.46 mm²), respectively, with variations of less than 5%, indicating minimal changes. This result confirms that CNT immersion does not significantly alter the macroscopic porous structure of the foam, ensuring the stability of its microstructure.

**
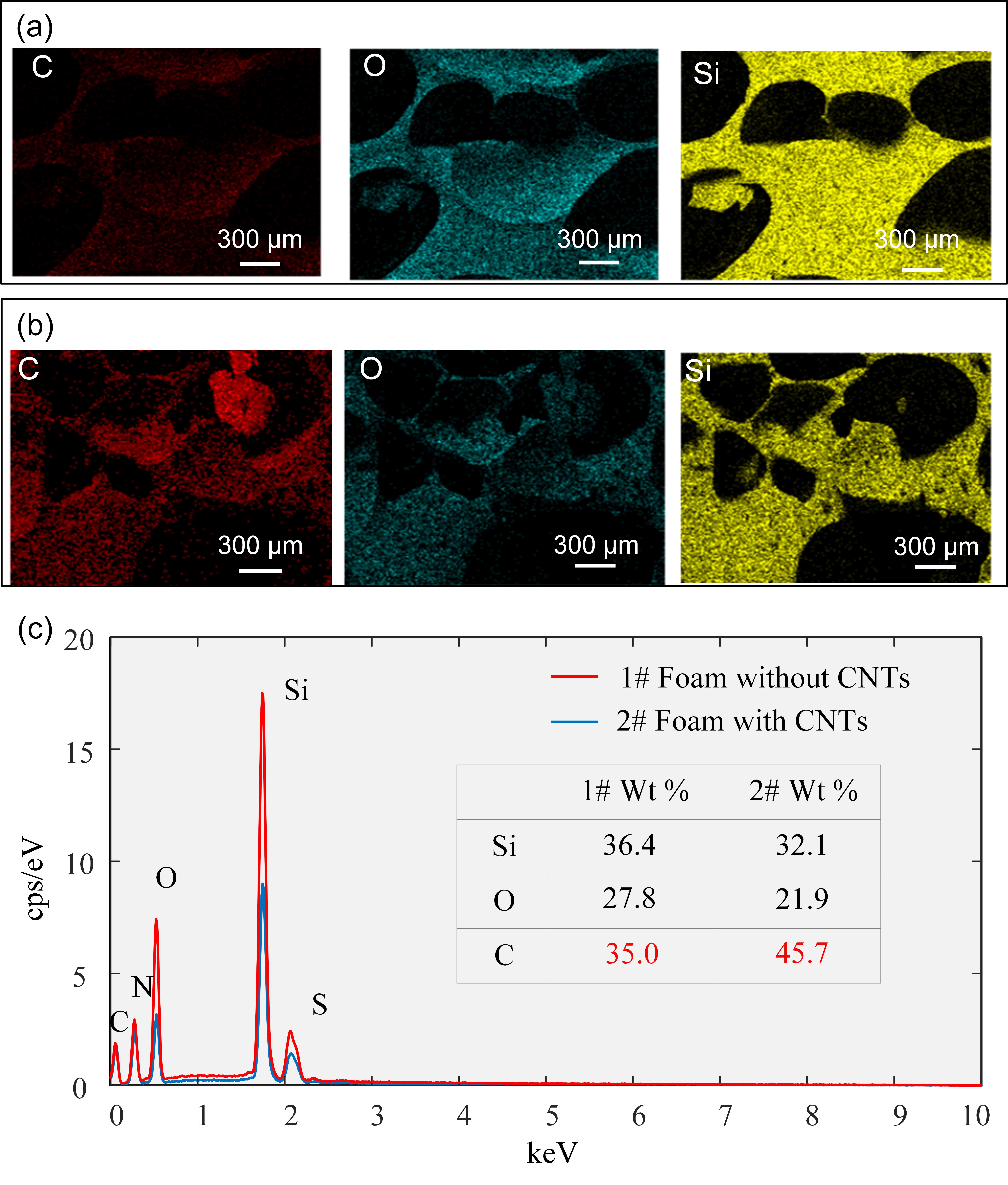
**

**Figure S3.** Analysis of elements content on the foam before and after CNTs were infiltrated.

Figures S3a and S3b illustrate the elemental distribution before and after CNT immersion, respectively. The carbon content in the sensor increases from 35.0% to 45.7%, as illustrated in Figures S3c. The carbon elements are uniformly distributed throughout the porous foam structure without noticeable local deposition, verifying the effectiveness of the CNTs immersion process in forming a conductive network. It is worth noting that the EDS analysis scans a flat plane, and some recessed areas within the foam's pores may not be detected. Thus, certain regions in Figures S3a and S3b appear darker in the images.


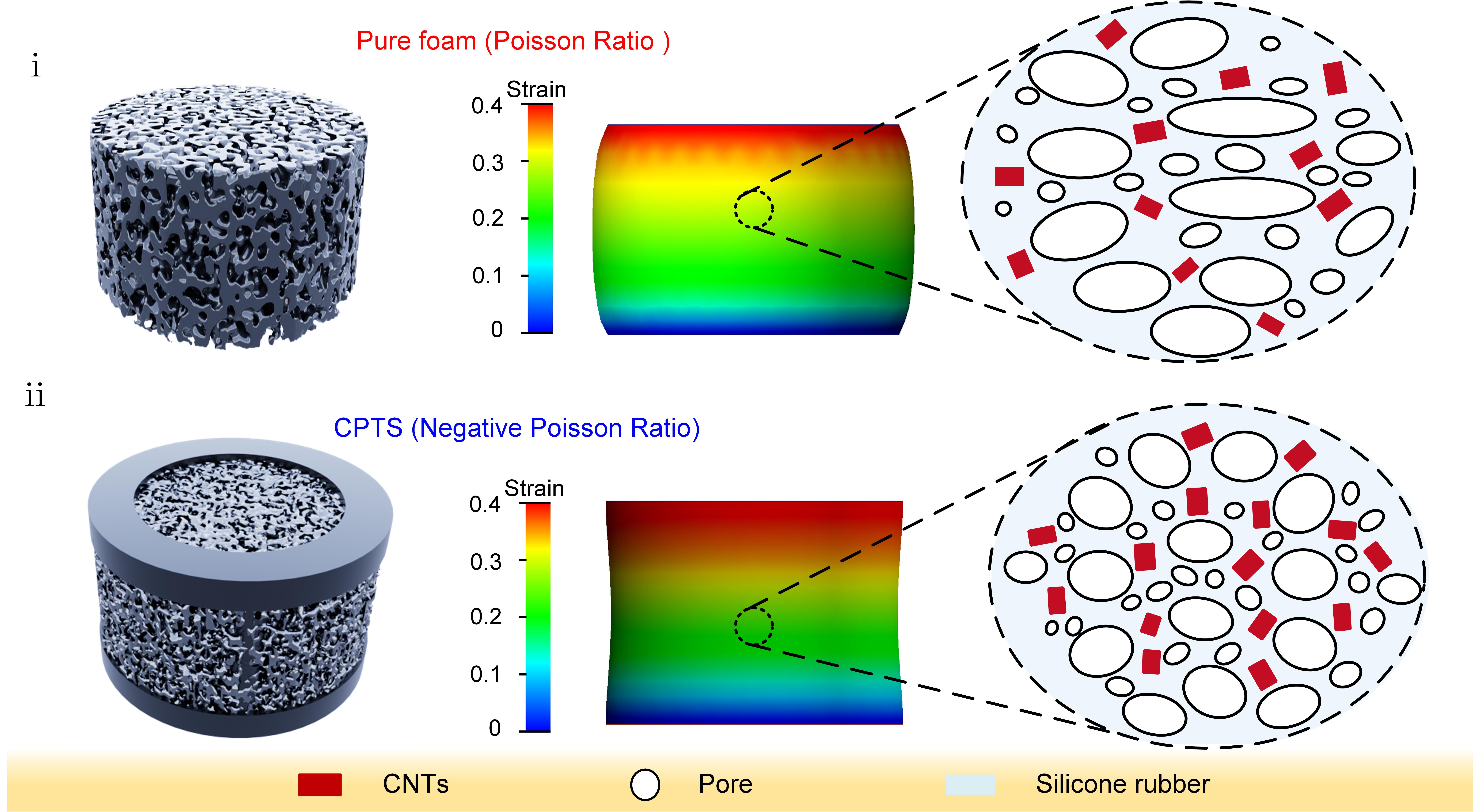


**Figure.S4** Microstructural deformation and conductive mechanism comparison between pure foam and CPTS under compression

As shown in Figure S4-i, conventional tactile sensors undergo positive Poisson’s ratio deformation under pressure, which hinders the rapid contact of carbon nanotube (CNT) particles and subsequently affects the sensor’s response speed. Therefore, by structural design achieving zero or negative Poisson’s ratio (Figure S4-ii), the contraction deformation of the tactile sensor can be effectively optimized. This allows CNTs to make rapid contact during compression while enabling a linear sensing range through resistance regulation.

Neglecting the influence of temperature on the material’s conductivity, the resistance of the conductive foam can be expressed as:

$R=\rho\frac{L}{V}=\rho\frac{L}{N_{0}e^{-\nu\varepsilon}}$ (1)

Where *R* represents the sensor resistance, $\rho$ is the resistivity, *L* and *V* denote the thickness and volume of the foam, respectively, *N*_0_​ is the initial number of conductive contact points, *ν* is the Poisson’s ratio, and $\varepsilon$ is the strain.

From a macroscopic perspective, the resistance *R* of the foam is related to its thickness *L* and volume *V*. During compression, the decrease in *V* reduces the resistance. From a microscopic perspective, the conductivity of the foam depends on the number of contact points *N* of the conductive particles. As compression increases *N*, the resistance further decreases.





**Figure S5.** a) Measuring system assembly. b) ESP32-S3 multi-channel signal acquisition and control circuit.

Figure S5 illustrates the circuit design of a multifunctional embedded system based on the ESP32-S3-WROOM-1-N8 module, integrating wireless communication, signal acquisition, and system control capabilities. The circuit is divided into several modules: 1) WIFI and Microprocessor Module: centered around the ESP32-S3-WROOM-1-N8, responsible for wireless communication and main control functions, with peripheral connections to power pins (VDD33 and GND) and multiple GPIO pins for system control and signal acquisition; 2) 5.5V to 3.3V Voltage Regulation Circuit: featuring an LD1117-3.3V voltage regulator to step down the 5.5V input to 3.3V, providing power to system components. It includes capacitors for filtering and an LED (D4) for power status indication; 3) Power Module: a simple design that provides a stable 5V input voltage; 4) Switch Module: comprising two push-button switches (BOOT and RST), connected to GPIO 0 and EN pins, respectively, for system boot and reset operations, facilitating debugging; 5) Detection Circuit Module: consisting of two LED circuits connected to GPIO pins through current-limiting resistors (R2 and R3), providing real-time system status indicators; 6) Signal Acquisition Circuit Module: consisting of multiple signal acquisition channels, with each channel connected to an independent GPIO pin through a resistor network. External signal inputs are supported via multiple 2x4 pin headers, enabling high-precision signal acquisition from multi-channel sensors. This design is modular, functionally clear, and suitable for embedded systems requiring WIFI communication, multi-channel signal acquisition, and flexible control.


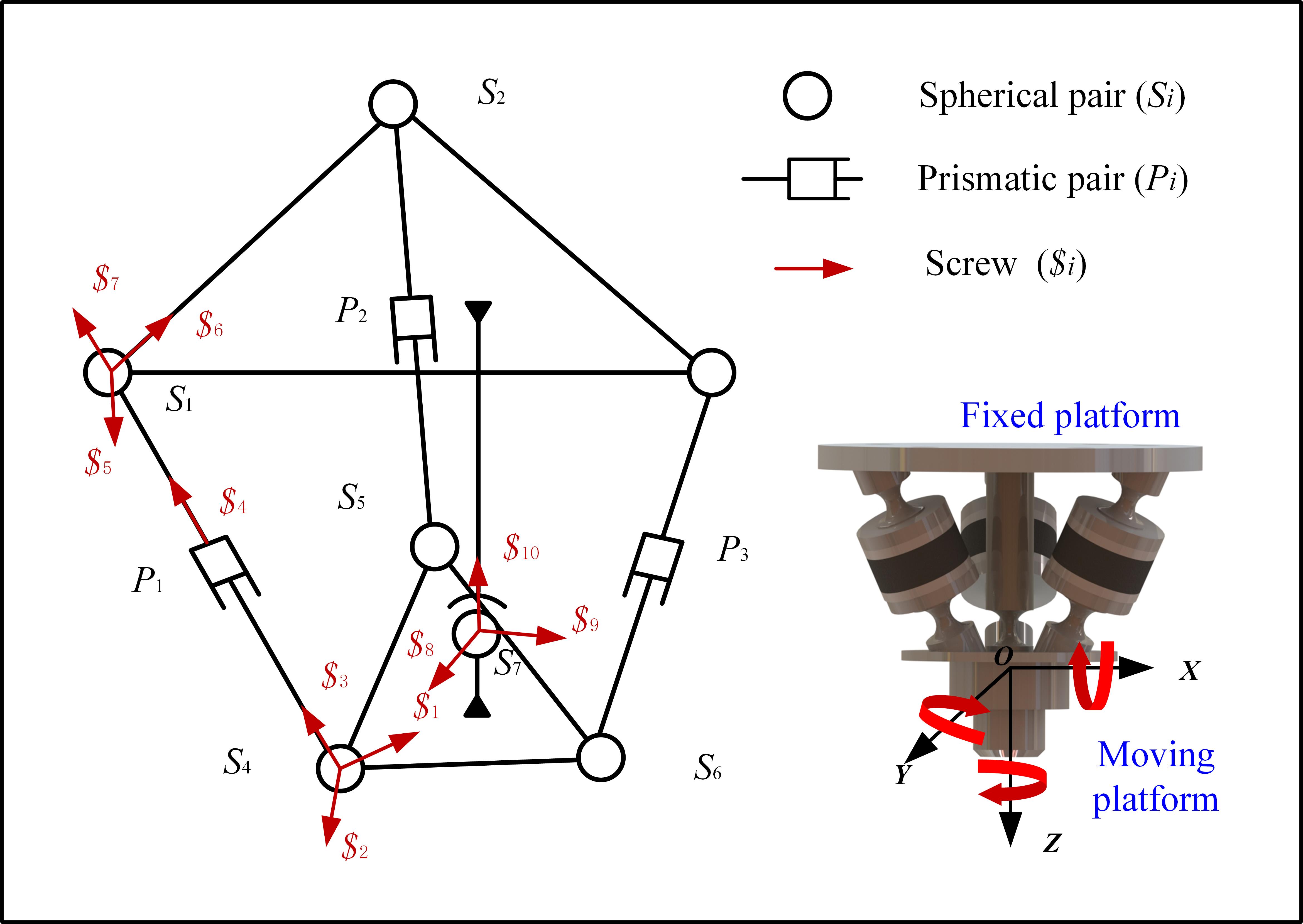


**Figure S6.** Kinematics analysis of three-dimensional torque decoupling measuring device.

Supplementary Notes

**Note S1. Design Principle of Wren elastomer.**

In the compression process, the lower end of the branch is fixed, and the upper end of the branch will rotate around the upper ring and fall with the upper platform. In order to achieve the effect of zero Poisson's ratio, the branch should always be in the interval between the inner ring and the outer ring during deformation without affecting the deformation of the central part of the inner ring. The branch structure should meet the following requirements:

The branch diameter $t$ should be less than the ring wall thickness $d$:

$d<t$ (1)

The projection of the branch on the Z-axis should meet the height requirements of the initial design:

$lsin\delta_{0}=h_{0}$ (2)

Where $l$ represents the branch length, if the branch length does not change during deformation, $\delta_{0}$ and $h_{0}$ represent the Angle between the axis of the branch and the *y* axis, and the initial height of elastomer, respectively.

The limit state of the branch in the process of compression and rotation is that the branch is tangent to the inner ring, which can be described as:

$\sqrt{r^{2}-\left( 0.5lcos\left( arcsin\left( \frac{h}{l} \right) \right) \right)^{2}}\geq r_{in}$ (3)

There can be no interference during rotation between branches:

$d>0.5lcos(arcsin(h/l))$ (4)

According to Equations (S1-S4), the structural constraints of Wren elastomer are obtained. Considering the size range of the preparation process, the design dimensions of Wren elastomer are shown in Table S1.

**Note S2. Derivation of the Mathematical Relationship Between Resistance Variation and External Force in CPTS-W.**

The initial vertical height of the structure is:

$H_{0}=l\cos\delta$ (1)

Each branch is modeled as a cantilever beam subjected to a compressive force. According to Euler–Bernoulli beam theory, the vertical displacement of the free end under axial load *F* is given by:

${\Delta H}_{0}=\frac{F\cos\delta}{EI}\cdot\frac{l^{3}}{6}$ (2)

Assuming the cross-sectional area *A* remains constant during compression, the volume becomes:

$V\left( F \right)=A\left( l\cos\delta-\frac{F\cos\delta}{EI}\cdot\frac{l^{3}}{6} \right)$ (3)

The conductive properties of conductive composites (insulating polymers filled with conductive materials) are analyzed using percolation theory. Considering the mechanical contact and tunneling effect, the resistance change can be described as:

$R=\frac{h^{2}\pi d}{Ae^{2}m\lambda}exp\left( \frac{2\lambda d}{h} \right)$ (4)

Where ***A*** represents the total contact area between the conductive particles within the foam, ***h*** is Planck's constant, ***m*** denotes the electron mass, ***d*** is the average spacing between conductive particles, ***λ*** represents the height of the insulating barrier, and e is the electron charge.

The relationship between the total volume of the material (𝑉), the total contact area (𝐴), and the average spacing between conductive particles (𝑑) is given by:

$\frac{d}{d_{0}}=\left( \frac{V_{0}}{V} \right)^{r_{1}}, \frac{A}{A_{0}}=\left( \frac{V_{0}}{V} \right)^{r_{2}}$ (5)

***r*_1_** and **𝑟_2_** are constants determined by the material properties, particle shape, and distribution density, which can be obtained through measurements of the foam material's volume changes under varying compression levels and microscopic characterization of the void area. Based on equations (4) and (5), the relationship between volume change and resistance can be derived as:

$R=\frac{h^{2}\pi d_{0}}{A_{0}e^{2}m\lambda}\left( \frac{V_{0}}{V} \right)^{\frac{r_{1}}{r_{2}}}exp\left( \frac{2\lambda d_{0}}{h} \right)\left( \frac{V_{0}}{V} \right)^{r_{1}}$ (6)

Based on Equations (3) and (6), the explicit expression for the resistance change under external force can be derived as:

$\begin{aligned} R\left( F \right)=\frac{h^{2}\pi d_{0}}{A_{0}e^{2}m\lambda}\left( V_{0} \right)^{\frac{r_{1}}{r_{2}}}\left( \left( A\left( l\cos\delta-\frac{F\cos\delta}{EI}\cdot\frac{l^{3}}{6} \right) \right)^{-\frac{r_{1}}{r_{2}}} \right) \\ exp\left( \frac{2\lambda d_{0}}{h} \right)\left( V_{0} \right)^{r_{1}}\left( \left( A\left( l\cos\delta-\frac{F\cos\delta}{EI}\cdot\frac{l^{3}}{6} \right) \right)^{{-r}_{1}} \right) \end{aligned}$ (7)

**Note S3. Calculation Methods for Sensor Sensitivity and Cyclic Stability Error.**

The sensor sensitivity 𝐾 is defined as:

$K=\frac{\Delta R/R_{0}}{\Delta P}$ (1)

Where $\Delta R/R_{0}$ represents the relative change in resistance, with $\Delta R$ and $R_{0}$represent the measured resistance and initial resistance, respectively. $\Delta P$ is the pressure change.

The cyclic repeatability error (**CR**) is used to evaluate the stability of the signal throughout the loading process.

$C_{R}=\frac{1}{n-1}\sum_{i=1}^{n} \left| \frac{R_{i+1}-R_{i}}{R_{i}} \right|\times100\%$ (2)

Where $R_{i}$ represents the resistance signal during the *i*-th loading cycle, and n is the total number of cycles. In the 50000 cycles test.

**Note S4. Derivation of the Mathematical Relationship Between Resistance Variation and External Force in CPTS-S.**

First, the conductive properties of conductive composites (insulating polymers filled with conductive materials) are analyzed using percolation theory. Considering the mechanical contact and tunneling effect, the resistance change can be described as:

$R=\frac{h^{2}\pi d}{Ae^{2}m\lambda}exp\left( \frac{2\lambda d}{h} \right)$ (1)

Where ***A*** represents the total contact area between the conductive particles within the foam, ***h*** is Planck's constant, ***m*** denotes the electron mass, ***d*** is the average spacing between conductive particles, ***λ*** represents the height of the insulating barrier, and e is the electron charge.

The relationship between the total volume of the material (𝑉), the total contact area (𝐴), and the average spacing between conductive particles (𝑑) is given by:

$\frac{d}{d_{0}}=\left( \frac{V_{0}}{V} \right)^{r_{1}}, \frac{A}{A_{0}}=\left( \frac{V_{0}}{V} \right)^{r_{2}}$ (2)

***r*_1_** and **𝑟_2_** are constants determined by the material properties, particle shape, and distribution density, which can be obtained through measurements of the foam material's volume changes under varying compression levels and microscopic characterization of the void area. Based on equations (7) and (8), the relationship between volume change and resistance can be derived as:

$R=\frac{h^{2}\pi d_{0}}{A_{0}e^{2}m\lambda}\left( \frac{V_{0}}{V} \right)^{\frac{r_{1}}{r_{2}}}exp\left( \frac{2\lambda d_{0}}{h} \right)\left( \frac{V_{0}}{V} \right)^{r_{1}}$ (3)

For an elastomer with central axial symmetry, the volume of the composite sensor can be expressed as:

$V=\sum_{i=1}^{n} V_{i}=\sum_{i=1}^{n} \frac{\pi}{n}\int_{0}^{H} \left( f\left( x \right) \right)^{2}dx$ (4)

Where *H* represents the current height of the composite material, and ***f*(*x*)** denotes the curve equation at the height *H*. Considering the large deformation characteristics of the branched structure, the elliptic integral method is employed to solve the curve equation of the parallel compliant mechanism. Here, different solving equations are derived for different mechanisms. Using an SS elastomer as an example, with a branch rod length of *L*, the internal moment of the flexible rod at a distance *x* from the free end along the rod direction is given by:

$M\left( x \right)=2F\left( L-x \right)cos\theta$ (5)

Based on the Bernoulli-Euler beam theory :

$\frac{d^{2}y}{d^{2}x}=\frac{2F\left( L-x \right)cos\theta}{EI}$ (6)

The result of the quadratic integral is :

$y=\frac{Fcos\theta}{EI}\left( -\frac{x^{2}}{3}+\frac{Lx^{2}}{2}+c_{1}x+c_{2} \right)$ (7)

For the fixed end, both the rotation angle and deflection are zero. Thus, when *x*=*L*, *y*=0 and $\frac{dy}{dx}=0$. Consequently, the deflection curve of the branch can be expressed as:

$y=\frac{Fcos\theta}{EI}\left( -\frac{x^{2}}{3}+\frac{Lx^{2}}{2}-\frac{L^{3}}{6} \right)$ (8)

Since all branches of the SS elastomer are identical, the envelope volume can be expressed using Equation (10) as:

$V\left( F \right)=\pi\left( -\frac{Fcos\theta}{24EI}L^{4}+\frac{Fcos\theta}{6EI}L^{3}-\frac{L^{3}}{6EI} \right)$ (9)

Figure 5b presents the theoretical results from Equation (15) alongside the deformation of the elastomer during compression. The regression coefficient R^2^ is 99.91%, indicating the validity of the theoretical results for the volume change of the SS elastomer.

Based on Equations (3) and (9), the explicit expression for the resistance change under external force can be derived as:

| $\begin{aligned} R\left( F \right)=\frac{h^{2}\pi d_{0}}{A_{0}e^{2}m\lambda}\left( V_{0} \right)^{\frac{r_{1}}{r_{2}}}\left( \pi\left( -\frac{Fcos\theta}{24EI}L^{4}+\frac{Fcos\theta}{6EI}L^{3}-\frac{L^{3}}{6EI} \right)^{-\frac{r_{1}}{r_{2}}} \right) \\ exp\left( \frac{2\lambda d_{0}}{h} \right)\left( V_{0} \right)^{r_{1}}\left( \pi\left( -\frac{Fcos\theta}{24EI}L^{4}+\frac{Fcos\theta}{6EI}L^{3}-\frac{L^{3}}{6EI} \right)^{{-r}_{1}} \right) \end{aligned}$ | (10) |
| --- | --- |

By adjusting the parameters of the compliant parallel mechanism, we aimed to improve the relationship between external force and resistance change, obtaining parameters with excellent linearity. Considering the material properties and manufacturing process characteristics, the final selected elastomer parameters are shown in Table S2.

**Note S5. Design and Kinematic Analysis of the 3D Force-Torque Decoupling Measurement Device**

Figure S6 depicts the composition of a three-dimensional torque decoupling measuring device. The degrees of freedom (DOF) of the three-dimensional torque decoupling measuring device are analyzed based on screw theory. To simplify the analysis, the three identical SPS branches are considered equivalent in structure, allowing the motion characteristics of the mechanism to be fully represented by examining a single branch. Specifically, the branch *S*_1_*P*_1_*S*_4_ is analyzed, where each spherical joint contributes three degrees of freedom, and the prismatic joint provides one degree of freedom. Accordingly, the motion screw system of the SPS branch is shown as follows:

$\left\{ \begin{matrix} \begin{matrix} {\$}_{1}=\left( 1 0 0;-0 0 0 \right) \\ {\$}_{2}=\left( 0 1 0;-0 0 0 \right) \end{matrix} \\ \begin{matrix} {\$}_{3}=\left( 0 0 1;-0 0 0 \right) \\ {\$}_{4}=\left( 1 0 0;-0 0 1 \right) \\ {\$}_{5}=\left( 0 1 0;-0 1 0 \right) \end{matrix} \\ \begin{matrix} {\$}_{6}=\left( 0 1 0;-1 0 0 \right) \\ {\$}_{7}=\left( 0 0 1;-0 0 0 \right) \end{matrix} \end{matrix} \right.$ (1)

The motion screw system contains 6 linearly independent screws, indicating that the DOF of this branch is 6. Thus, the SPS branch imposes no constraints on the parallel mechanism. Next, for the S branch, its motion screw system is expressed as:

$\left\{ \begin{matrix} {\$}_{7}=\left( 1 0 0;-0 l 0 \right) \\ {\$}_{8}=\left( 0 1 0;-l 0 0 \right) \\ {\$}_{9}=\left( 0 0 1;-0 0 0 \right) \end{matrix} \right.$ (2)

Where *l* represents the length of *S* branch. By calculating the reciprocal product, the constraint screw system of the S branch can be derived as:

$\left\{ \begin{matrix} {{\$}^{r}}_{7}=\left( 1 0 0;-0 l 0 \right) \\ {{\$}^{r}}_{8}=\left( 0 1 0;-l 0 0 \right) \\ {{\$}^{r}}_{9}=\left( 0 0 1;-0 0 0 \right) \end{matrix} \right.$ (3)

The *S* branch constrains the movement of the parallel mechanism's moving platform along the x, y, z directions. Consequently, the moving platform has three degrees of freedom, which are rotations around the x, y, z axes.

Further verification is conducted based on the modified Grübler-Kutzbach (G-K) formula, expressed as follows:

$M=d\left( n-g-1 \right)+\sum_{i=1}^{g} f_{i}+v+\xi=3$ (4)

where 𝑀 represents the DOF of the mechanism, 𝑛 is the total number of components including the base, 𝑔 denotes the number of kinematic pairs, and 𝑓_𝑖_ refers to the DOF of the 𝑖-the kinematic pair. Additionally, 𝑣 accounts for the redundant constraints within the mechanism, 𝜉 represents the local degrees of freedom present in the mechanism, and 𝑑 is the order of the mechanism. In the case of the three SPS branches, there are 3 local degrees of freedom, 𝜉=3.

Finally, the three-dimensional torque decoupling measuring device possesses three rotational degrees of freedom.

Supplementary Tables

**Table S1.** Wren elastomer Structural parameters

| $l$ | $d$ | $t$ | $r$ | $\delta$ |
| --- | --- | --- | --- | --- |
| 10.20 mm | 2.00 mm | 3.00 mm | 17.00 mm | 5° |

**Table S2.** SS elastomer parameters

| $L$ | $I$ | $E$ | $r_{1}$ | $r_{2}$ |
| --- | --- | --- | --- | --- |
| 5.24 mm | 0.17 mm^4^ | 6.10 MPa | 7.21 | 0.94 |
| $\theta$ | $\lambda$ | $d_{0}$ | $V_{0}$ | $A_{0}$ |
| 45 ° | 0.30 eV | 120 2 μm | 50.27 mm^3^ | 148 mm^2^ |

**Table S3**. Properties comparison of the reported sensors and that in this work.

| Range | Sensitivity | Response  Time | Cycle | Cycle  Error | Reference |
| --- | --- | --- | --- | --- | --- |
| 0-150 kPa | 0.32 kPa⁻¹ (0-8 kPa) | 120/80 ms | 250 | / | [1] |
| 0-584.4 kPa | GF=0.8 | 150 ms | 10000 | 7.47% | [2] |
| 0-14 kPa | 28.62 kPa⁻¹ (0-14 kPa) | 37/14 ms | 2600 | 9.43% | [3] |
| 0-10 kPa | 0.025 kPa⁻¹ (0-6 kPa) | 60 ms | 5000 | / | [5] |
| 0-80 kPa | 0.151 kPa⁻¹ (0-2 kPa)  0.035 kPa⁻¹ (10-40 kPa) | 100 ms | 1000 | / | [6] |
| 0-171 kPa | 2.456 kPa⁻¹ (0-10 kPa)  0.106 kPa⁻¹ (28-171 kPa) | 225/50 ms | 2000 | / | [8] |
| 0.5 Pa-200 kPa | 0.583 kPa⁻¹ (0-1.2 kPa) | 40/45 ms | / | 11.8% | [9] |
| 0-500 kPa | 2.155 kPa⁻¹ (0-2 kPa)  0.022 kPa⁻¹ (200-500 kPa) | 248/90 ms | 2500 | / | [12] |
| 0-300 kPa | 1126.96 kPa⁻¹ (20-120 kPa) | 25/50 ms | 6000 | 0.88% | [13] |
| 0-250 kPa | 0.746 kPa⁻¹ (50-250 kPa) | 120 ms | 2000 | / | [15] |
| 0-100 kPa | 0.213 kPa⁻¹ (0-25 kPa)  0.349 kPa⁻¹ (25-110 kPa) | 50 ms | 1000 | 9.56% | [16] |
| 0-450 kPa | 0.008 kPa⁻¹ (0-50 kPa)  0.0012 kPa⁻¹ (98-423 kPa) | 48/39 ms | 50000 | 0.12% | This work |

[1] Y. Tian, D.-Y. Wang, Y.-T. Li, H. Tian, Y. Yang, T.-L. Ren, IEEE Trans. Electron Devices 2020, 67, 2153.

[2] Y. Zhai, Y. Yu, K. Zhou, Z. Yun, W. Huang, H. Liu, Q. Xia, K. Dai, G. Zheng, C. Liu, C. Shen, Chemical Engineering Journal 2020, 382, 122985.

[3] X. Cao, J. Zhang, S. Chen, R. J. Varley, K. Pan, Adv Funct Materials 2020, 30, 2003618.

[4] Y. Cheng, L. Li, Z. Liu, S. Yan, F. Cheng, Y. Yue, S. Jia, J. Wang, Y. Gao, L. Li, Research 2022, 2022, 9843268.

[5] H. Zhu, H. Luo, M. Cai, J. Song, Advanced Science 2024, 11, 2307693.

[6] A. Huang, Z. Yang, Y. Zhu, B. Tan, Y. Song, Y. Guo, T. Liu, X. Peng, Applied Surface Science 2023, 618, 156661.

[7] S. Song, C. Zhang, W. Li, J. Wang, P. Rao, J. Wang, T. Li, Y. Zhang, Nano Energy 2022, 100, 107513.

[8] X. Guo, W. Hong, Y. Zhao, T. Zhu, H. Li, G. Zheng, J. Wang, G. Tang, J. Cao, Y. Wang, J. Yang, H. Zhang, D. Zhou, R. Feng, D. Xu, Q. Hong, Y. Xu, Composites Part A: Applied Science and Manufacturing 2022, 163, 107240.

[9] Y. Zhao, X. Guo, W. Hong, T. Zhu, T. Zhang, Z. Yan, K. Zhu, J. Wang, G. Zheng, S. Mao, K. Wang, Y. Wang, C. Jin, G. Tang, S. Shao, Y. Xia, G. Xing, Q. Hong, Y. Xu, J. Wu, Composites Science and Technology 2023, 231, 109837.

[10] C. Mahata, H. Algadi, J. Lee, S. Kim, T. Lee, Measurement 2020, 151, 107095.

[11] M. Chao, L. He, M. Gong, N. Li, X. Li, L. Peng, F. Shi, L. Zhang, P. Wan, ACS Nano 2021, 15, 9746.

[12] Z. Feng, Q. He, X. Wang, Y. Lin, J. Qiu, Y. Wu, J. Yang, ACS Appl. Mater. Interfaces 2023, 15, 6217.

[13] Y.-D. Tang, P. Wang, G.-X. Li, G.-Y. Wang, W. Yu, C.-Z. Meng, S.-J. Guo, Adv Eng Mater 2023, 25, 2200814.

[14] J. Lin, Y. Ai, W. Li, Y. Peng, Y. Liu, A. He, H. Nie, Composites Science and Technology 2023, 237, 110005.

[15] X. Chen, D. Zhang, H. Luan, C. Yang, W. Yan, W. Liu, ACS Appl. Mater. Interfaces 2023, 15, 2043.

[16] Q. Hong, T. Liu, X. Guo, Z. Yan, W. Li, L. Liu, D. Wang, W. Hong, Z. Qian, A. Zhang, Z. Wang, X. Li, D. Wang, Z. Mai, Y. Zhao, F. Yan, G. Xing, Sensors and Actuators B: Chemical 2024, 404, 135255.

**Video S1** Compression deformation of the Sarrus elastomer

**Video S2** Compression deformation of the Wren elastomer

**Video S3** Application test: Human health monitoring

**Video S4** Application test: Gesture-based Human-Machine Interaction

**Video S5** Application test: Torque Sensing
